# Novel lipometabolism biomarker for chemotherapy and immunotherapy response in breast cancer

**DOI:** 10.1186/s12885-022-10110-8

**Published:** 2022-10-01

**Authors:** Lei Zhang, Risheng She, Jianlin Zhu, Jin Lu, Yuan Gao, Wenhua Song, Songwang Cai, Lu Wang

**Affiliations:** 1 Department of Gastrointestinal Surgery, the First Affiliated Hospital of Jinan University, Guangzhou, 510632 China; 2 Institute of Precision Cancer Medicine and Pathology, School of Medicine, Jinan University, Guangzhou, 510632 China; 3 Department of Oncology Surgery, the Second Affiliated Hospital of Bengbu Medical College, Bengbu, 233080 Anhui Province China; 4 Department of Emergency, Dongguan People’s Hospital, Dongguan, 523000 China; 5 Laboratory of Computational Medicine and Intelligent Health, Bengbu Medical College, Bengbu, 233030 Anhui Province China; 6 Department of Medical Ultrasound, the Second Affiliated Hospital of Bengbu Medical College, Bengbu, 233080 Anhui Province China; 7 Department of Thoracic Surgery, The First Affiliated Hospital of Jinan University, Guangzhou, Guangdong 510630 P. R. China

**Keywords:** Lipometabolism, Tumor microenvironment, Immunotherapy, Chemotherapy, Breast cancer

## Abstract

Emerging proof shows that abnormal lipometabolism affects invasion, metastasis, stemness and tumor microenvironment in carcinoma cells. However, molecular markers related to lipometabolism have not been further established in breast cancer. In addition, numerous studies have been conducted to screen for prognostic features of breast cancer only with RNA sequencing profiles. Currently, there is no comprehensive analysis of multiomics data to extract better biomarkers. Therefore, we have downloaded the transcriptome, single nucleotide mutation and copy number variation dataset for breast cancer from the TCGA database, and constructed a riskScore of twelve genes by LASSO regression analysis. Patients with breast cancer were categorized into high and low risk groups based on the median riskScore. The high-risk group had a worse prognosis than the low-risk group. Next, we have observed the mutated frequencies and the copy number variation frequencies of twelve lipid metabolism related genes LMRGs and analyzed the association of copy number variation and riskScore with OS. Meanwhile, the ESTIMATE and CIBERSORT algorithms assessed tumor immune fraction and degree of immune cell infiltration. In immunotherapy, it is found that high-risk patients have better efficacy in TCIA analysis and the TIDE algorithm. Furthermore, the effectiveness of six common chemotherapy drugs was estimated. At last, high-risk patients were estimated to be sensitive to six chemotherapeutic agents and six small molecule drug candidates. Together, LMRGs could be utilized as a de novo tumor biomarker to anticipate better the prognosis of breast cancer patients and the therapeutic efficacy of immunotherapy and chemotherapy.

**Supplementary Information:**

The online version contains supplementary material available at 10.1186/s12885-022-10110-8.

## Introduction

Breast cancer (BRCA) has become the most common malignancy and the second leading cause of cancer-related deaths globally [1]. At present, there are some therapies for breast cancer such as classical chemotherapy, radiotherapy, endocrine therapy and other targeted therapies. However, some patients can occur drug resistance [2]. At the same time, immunotherapy is relatively rare [3]. It may be related to the immune microenvironment of BRCA [4–6]. Thus, it is imperative to uncover new therapeutic biomarkers to guide clinical treatment.

Lipometabolism has been proved to have a significant relationship with invasion, metastasis and cancer stemness [7]. Altered lipometabolism is among the most remarkable metabolic changes in cancer. The enhanced synthesis and uptake of lipids contribute to the rapid growth of cancer cells and tumor progression. Lipids are a highly complex group of biomolecules that not only comprise the structural substratum of biological membranes but also act as signaling molecules and energy sources. It is known as the cancer metabolic reprogramming [8]. Some studies have shown that tumor cells can achieve immune escape through metabolic reprogramming [9]. For expamble, lipometabolism is the process in which breast cancer cells can be better growth [10, 11]. Meanwhile, lipometabolism reprogramming can also induce tumor resistance in chemotherapy and immunotherapy [12–14]. Through cellular and animal experiments, some relevant markers of lipid metabolism have been identified in breast cancer. [15]. However, it’s rare that the markers of lipid metabolism were screened by large clinical samples. Nevertheless, there are no biomarkers for lipometabolism-related chemotherapy and immunotherapy in BRCA.

In this study, a riskScore of the lipometabolism-related gene signature consisting of twelve genes was constructed by univariate Cox regression and LASSO regression analysis. In addition, these twelve genes were analyzed for tumor mutation burden and copy number variation. This prognostic model accurately predicted the overall survival and reflected the efficacy of TIME and immunotherapy in BRCA. And we examined the association between risk groups and BRCA stemness and successfully predicted IC50 scores for chemotherapy in both high- and low- risk groups. In addition, we proposed a treatment strategy for the high-risk group. In conclusion, the results of this study can help clinicians and oncologists to predict the breast cancer prognosis and the efficacy of chemotherapy and immunotherapy.

## Materials and methods

### Patients and clinical specimens

We searched The Cancer Genome Atlas (TCGA) breast cancer cohort for RNA-Seq, single nucleotide variants and copy number variants in the dataset (https://nci.nih.gov/tcga/) excluding patients with no clinical information. A total of 1064 samples were included in the study, with 1014 cancer specimens and 60 normal samples. We annotated transcripts with gene transfer format (GTF) documents obtained from Ensembl.

### Identification of lipometabolism related DEGs and functional richness analysis

The limma package in R V4.1.1 (https://www.r-project.org; |log2fold change|> 1, (FDR) < 0.05) analyzes DEGs, the volcano map and the differential gene heat map use the R package respectively Ggplot2 and heatmap packages in the. Then provide GO and Kyoto Encyclopedia of Genes and Genomes (KEGG) enrichment analysis (adjPvalue < 0.05) through the clusterProfiler package [16].

### Univariate Cox analysis and construction of prognostic model

Using lipometabolism differential gene data, the survival package is used for univariate Cox regression analysis. The least absolute shrinkage and selection operator (LASSO) regression algorithm for feature selection, using 10-fold cross-validation, the above analysis uses the R software package glmnet. For Kaplan–Meier curves, *p*-values and hazard ratio (HR) with 95% confidence interval (CI) were generated by log-rank tests and univariate Cox proportional hazards regression. All analytical methods above and R packages were performed using R software version 4.0.5 (The R Foundation for Statistical Computing, 2020). *p* < 0.05 was considered as statistically significant.

### Estimation of STromal and Immune cells in MAlignant Tumour tissues using Expression data (ESTIMATE)

The ESTIMATE algorithm-generated matrix and immune scores to estimate the level of infiltrating matrix and immune cells in BRCA tissue and tumor purity through expression profiles. Then, we used the Wilcoxon rank-sum test to compare the differences in tumor purity, stroma, and immune scores between the high and low risk groups.

### Screening chemotherapy agents and predicting the effective response of Immunotherapy

Screening of chemotherapeutic agents in the high- and low-risk groups was performed with the R package "pRRophetic". Immunophenoscore (IPS) of BRCA patients was derived from the Cancer Immunology Atlas (TCIA, https://tcia.at/patients). The patient's IPS was obtained without prejudice by considering four types of immunogenicity determinants: effector cells, immunosuppressive cells, MHC molecules, and immunomodulators. This step is performed by evaluating gene expression in the four cell types. IPS is calculated based on the z-score representing the gene expression in the cell type in the range of 0–10. A higher IPS score is positively correlated with increased immunogenicity. Meanwhile, Tumor Immune Dysfunction and Exclusion (TIDE) algorithm predicted ICB response and evaluates immune escape ability (http://tide.dfci.harvard.edu/login/).

### The prediction of potential small molecule agents in BRCA patients

The Connectivity Map (CMap) database (https://portals.broadinstitute.org/cmap/) was used to predict potential drugs. The full range of up-and down-regulated overlapping genes was submitted to the CMap database to predict drugs that might induce or reverse the biological processes that characterize the expression of specific genes in BRCA. Enrichment scores were calculated from -1 to 1. Enrichment scores between -1 and 0 indicated that the drug might reverse gene expression (a candidate for BRCA). In contrast, enrichment scores between 0 and 1 indicated that the drug might induce gene expression. *p*-values < 0.05 were considered statistically significant. Finally, 2D structural graphs of these drug candidates were obtained from PubChem (https://pubchem.ncbi.nlm.nih.gov/).

### Construction of Nomogram based on prognostic model

The "rms" package in R builds a nomogram based on overall survival (OS) with independent prognostic factors. Use the AUC value to test the ability of the Nomogram to distinguish survival. Construct a calibration curve of the Nomogram to test the 1, 3 and 5-year survival probabilities based on the Nomogram and actual observations.

### Statistical analysis

Statistical analysis is performed by R (version 4.1.1). The Wilcoxon rank-sum test presents comparisons between the two groups, while the Kruskal–Wallis test assesses multiple comparisons. The survminer package determines the demarcation point of each subgroup in R. The Kaplan–Meier curve of OS analysis was presented between different subgroups, and then the log-rank test was performed. Multivariate cox regression analysis is used to evaluate the association between OS and clinicopathological characteristics and risk scores. The Forestplot package visualizes these in R. AUC depicts 1, 3, and 5-year survival rates and is used to assess the predictive power of risk scores. Bonferroni's test corrects the *P*-value. *P* < 0.05 on both sides was considered statistically significant.

## Results

### Differential gene expression of lipometabolism-related genes in breast cancer

A total of 751 LMRGs were identified from previous studies for enrollment in this study (Table S1). To determinate the differential expression levels of LMRGs in breast cancer and normal tissues from the TCGA dataset (TCGA_BRCA), we identified 294 differential genes (|logFC|> 0.5, *p* < 0.05, Table S2). As the volcano map revealed, 136 genes were upregulated and 158 genes were downregulated in breast cancer (Figure S1A). Meanwhile, we performed the relationship between differential genes and the overall survival of BRCA patients in the TCGA dataset. Univariate Cox regression indicated thirty genes were significantly associated with independent prognostic risk factors (Figure S1B, *p* < 0.05).

### Construction and verification of riskSore of lipometabolism-related genes

**Fig. 1:**
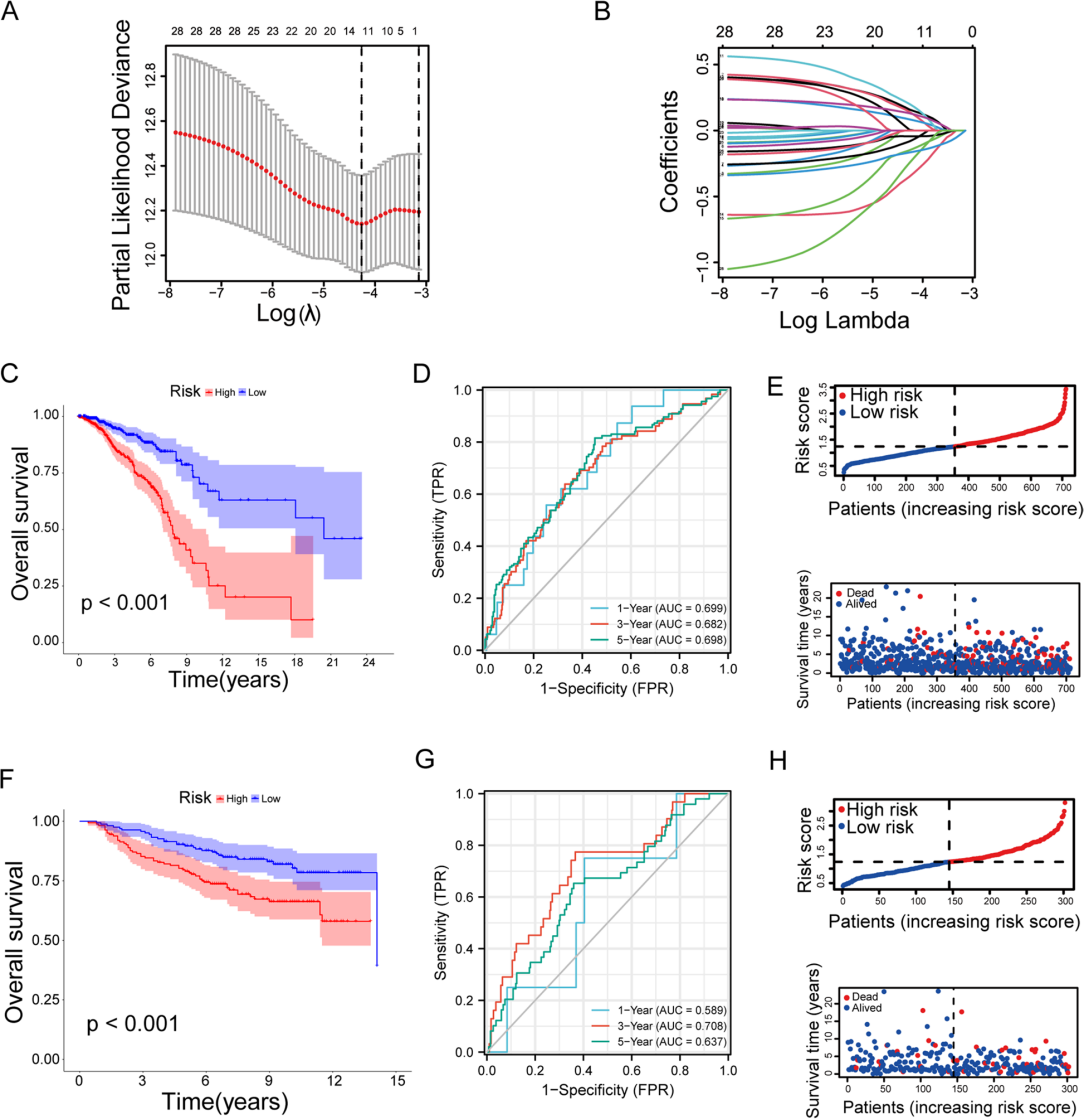
Construction and verification of prognostic model of lipometabolism-related genes.** A** Tuning parameter (λ) selection cross‐validation error curve. **B** Distribution of LASSO coefficients for the 141 survival-related LMRGs. **C** Kaplan–Meier curve for internal training set. **D** 1, 3 and 5 year time dependent ROC curves for internal training set. **E** Risk score and survival time based on the LMRGs of internal training cohort. **F** Kaplan–Meier curve for external validation sets. **G** 1, 3 and 5 year time dependent ROC curves for external validation sets. **H** Risk score and survival time based on the LMRGs of external validation sets

To construct a risk score for lipid metabolism associated with BRCA, we performed LASSO regression analysis on the thirty genes mentioned above, generating signatures for twelve genes (ABCA1, PIK3CA, OSBPL1A, ACSL1, APOA5, NDUFAB1, ENPP6, PLA2G2D, SRD5A3, PLEKHA4, SRD5A2, CEBPD) (Fig. 1A-B). The risk score for each patient is calculated through the formula: riskScore = $\sum_{i=1}^{n}\beta i\chi i$. We categorized BRCA patients into high-and low- risk groups based on the median risk score. We used TCGA_BRCA as the internal training set, Kaplan–Meier analysis showed that high-risk patients had a poorer prognosis (Fig. 1C, *p* < 0.001). And the 1-, 3-, and 5-year AUC values were 0.699,0.682 and 0.698 in the TCGA_BRCA (Fig. 1D). Risk curves showed that riskScore was positively correlated with risk values of BRCA patients (Fig. 1E). Further, we used GEO dataset (GSE20685) as the validation cohort. Kaplan–Meier analysis showed that high-risk patients had a poorer prognosis (Fig. 1F, *p* < 0.01) and the 1-, 3-, and 5-year AUC values were 0.589, 0.708 and 0.637 (Fig. 1G). Risk curves showed that riskScore was positively correlated with risk values for BRCA patients in the validation cohort (Fig. 1H).

### Association between riskScore and clinicopathological features of BRCA

**Fig. 2:**
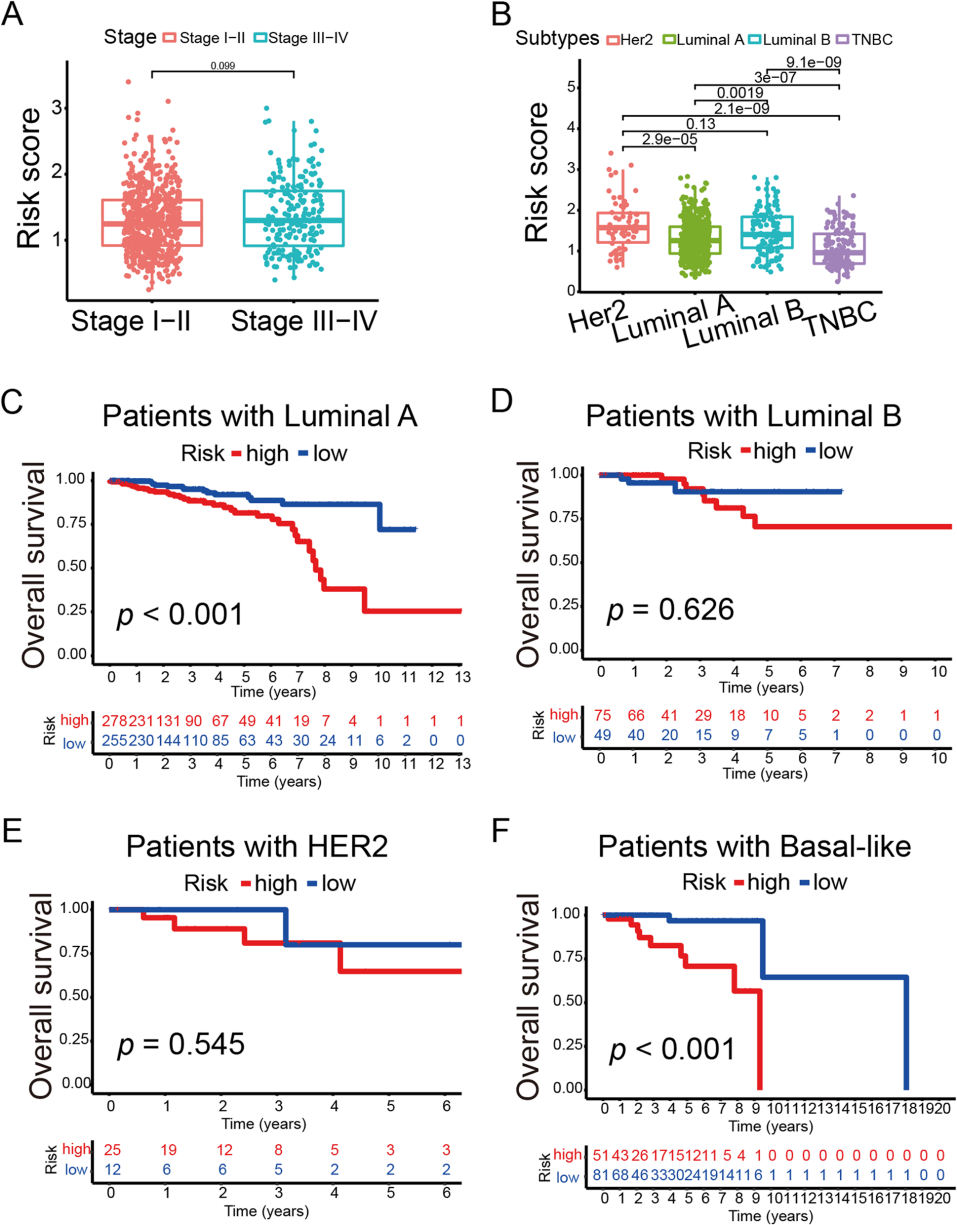
The correlation of riskScore with patients’ clinicopathological characteristics. **A** Association between clinical stage and riskScore. **B** Association between molecular subtypes and riskScore. **C** Survival rates of Luminal A breast cancer in high and low risk groups. **D** Survival rates of Luminal B breast cancer in high and low risk groups. **E** Survival rates of HER2 breast cancer in high and low risk groups. **F** Survival rates of Basal-like breast cancer in high and low risk groups

To clarify the relationship between riskScore and the clinicopathological features and molecular subtypes of BRCA, we observed that high riskScore was positively correlated with age (Figure S2A-D, *p* < 0.01), but not statistically significant with T stage, N stage and M stage (*p* > 0.05). The risk scores for the late stage (stage III/IV) were not statistically different from those for the early stage (stage I/II) (Fig. 2A, *p* = 0.099). Due to the classification of BRCA into Luminal A, Luminal B, HER2-positive and triple-negative types based on ER, PR and HER2 expression, we further elaborated on the relevance of riskScore to the molecular subtypes of BRCA. Interestingly, riskScore was significant difference in ER and HER2 states (Figure S3A-C, *p* < 0.05). Meanwhile, in HER2-positive riskScore was significant different from Luminal A subgroup (Fig. 2B, *p* < 0.05). We also validated age, ER and HER2 in the GSE6130 dataset and the results showed that the risk scores were higher in ER negative group, but no significant difference in age or HER2 group. (Figure S3D-F, *p* < 0.05). Further, we revealed the clinicopathological characteristics between the high-risk and low-risk groups. According to the age, the high-risk group had a lower OS (Figure S4A-B, *p* < 0.05). BRCA patients had worse OS in the high riskScore model on the clinical stage I/II (Figure S5A-B, *p* < 0.05). In terms of ER, PR and HER2, the prognosis of high-risk group was worse in the ER-positive/negative, PR-positive/negative and HER2-negative BRCA subgroups from TCGA-BRCA (Figure S6A-F, *p* < 0.05). Meanwhile, we used GSE6130 dataset and found the prognosis was no statistical significance in different subgroups (Figure S6G-J). Further, in the case of molecular subtypes, the prognosis was worse in the high-risk group in Luminal A and TNBC subtypes while no statistical significance was showed in HER2 and Luminal B subtypes (Fig. 2C-F, *p* < 0.05). We also plotted the relationship between risk score and molecular type and analyzed survival prognosis in the GSE6130 dataset (Figure S7).

### Prognostic model based on Nomogram to foresee the survival of BRCA patients

**Fig. 3:**
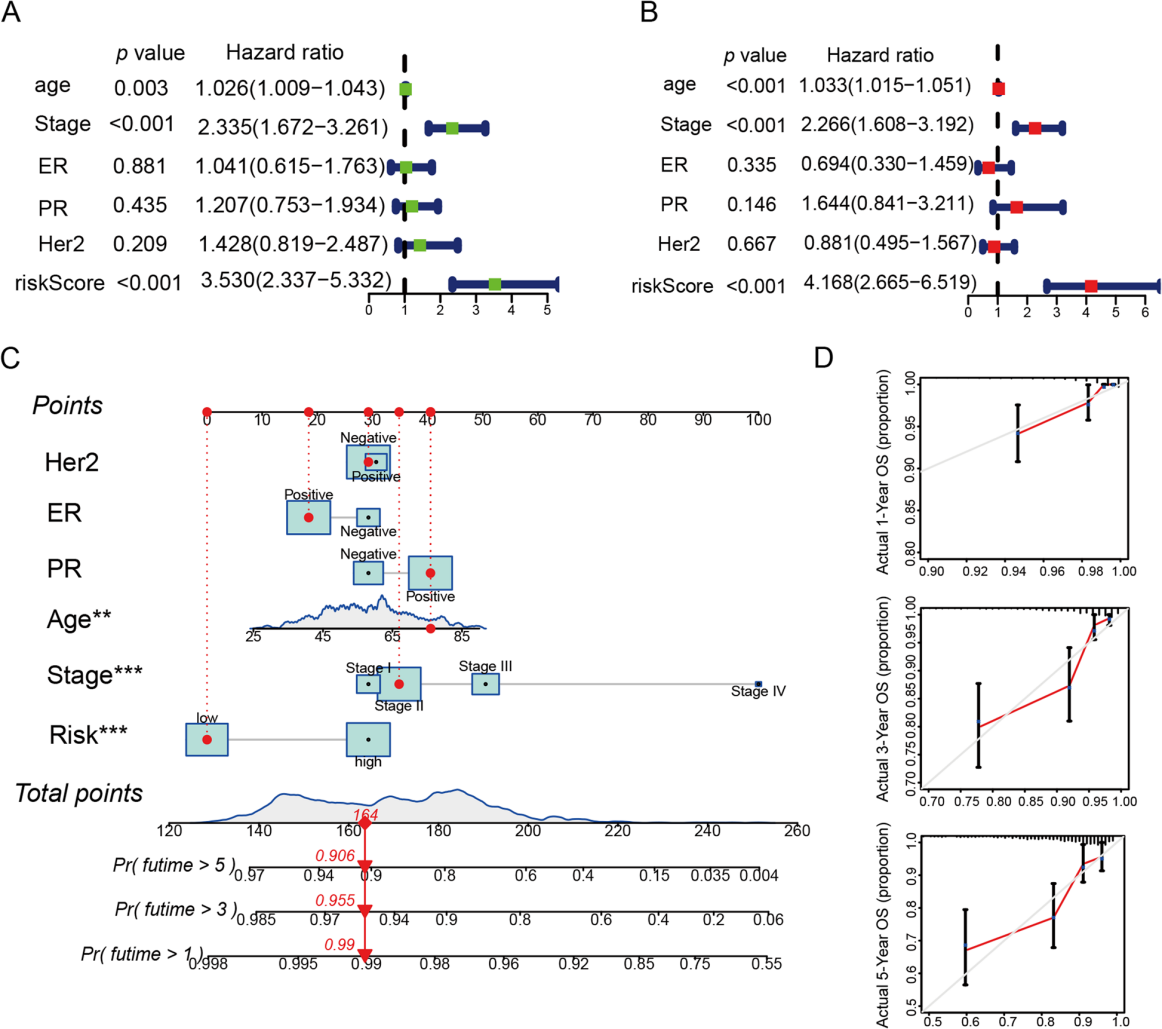
Prognostic model based on Nomogram to foresee the survival of BRCA patients. **A** Forest plot summary of univariate regression analyses of riskScore and clinicopathological characteristics in TCGA-BRCA cohort. **B** Forest plot summary of multivariate regression analyses of riskScore and clinicopathological characteristics in TCGA-BRCA cohort. **C** Nomograms for predicting the probability of patient mortality at 1-,3- or 5-year OS based on riskScore. **D** Calibration curves of the nomogram for predicting the probability of OS at 1-,3-, and 5-years

Univariate and multivariate Cox regression demonstrated that the riskScore was an independent predictor for poorer overall survival (Fig. 3A-B). Therefore, we constructed the Nomogram based on the riskScore model (Fig. 3C) and the calibration diagram was listed in Fig. 3D. Meanwhile, the Nomogram was constructed with the GSE6130 dataset (Figure S8). These data suggested that the riskScore-based Nomogram might serve as a robust tool for the prediction of survival in patients with BRCA.

### Transcriptome, single nucleotide mutations and copy number variations in LMRGs of breast cancer

**Fig.4:**
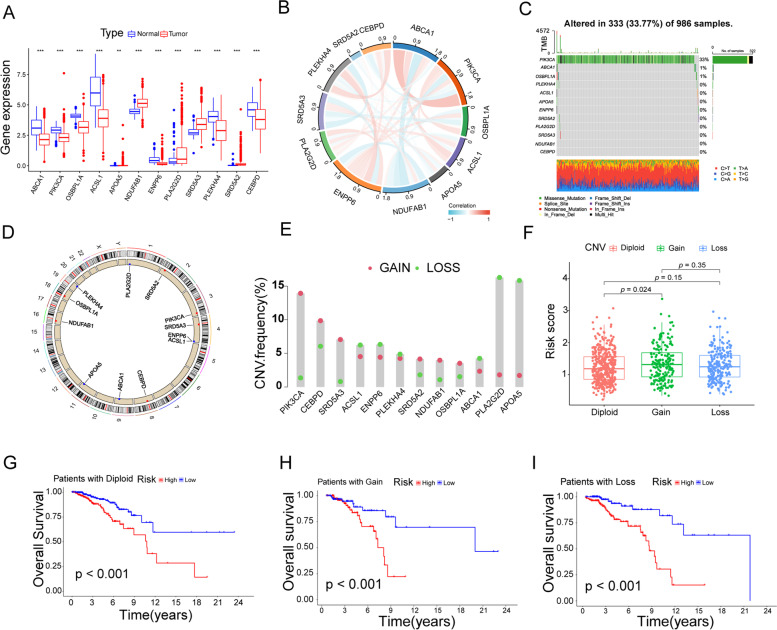
Transcriptome, single nucleotide mutations and copy number variations in LMRGs of breast cancer. **A** Variance analysis of twelve LMRGs in breast cancer Waterfall diagram of LMRGs of breast cancer. **B** Chord diagram of the interrelationship of the twelve LMRGs in breast cancer. **C** Waterfall plot of twelve LMRGs mutations in breast cancer. **D** LMRGs copy number variation circle map of breast cancer. **E** Copy number variation frequency of LMRGs in breast cancer. **F** Correlation of LMRGs expression levels with different CNV patterns. **G**-**I** KM survival curve of patients with diploid,gain and loss LMRGs in high and low risk groups

To ascertain the relationship between genetic alterations and interactions of LMRGs in BRCA, we first observed the differential expression of these twelve genes at the transcriptome level in cancerous and paraneoplastic tissues (Fig. 4A). Naturally, we appraised the interaction network diagram among the twelve LMRGs (Fig. 4B). Subsequently, we assessed the prevalence of somatic mutations (SNVs) and copy number variants (CNVs). 333 of the 986 samples were detected as carrying mutations in the LMRGs, with the highest mutation frequency being PI3KCA (Fig. 4C). The location of CNV alterations on the chromosomes of the 12 LMRGs was shown in Fig. 4D. And the frequency of CNV mutations was shown in Fig. 4E. We looked at the relationship between CNV characteristics and risk scores. The results showed that the Gain group had a higher risk score than the diploid group (Fig. 4F, *p* < 0.024). Survival differences were found in the diploid, Gain and Loss groups in both the high and low risk groups (Fig. 4G-I, *p* < 0.05).

### Relationship between prognosis models and immune microenvironment

**Fig. 5:**
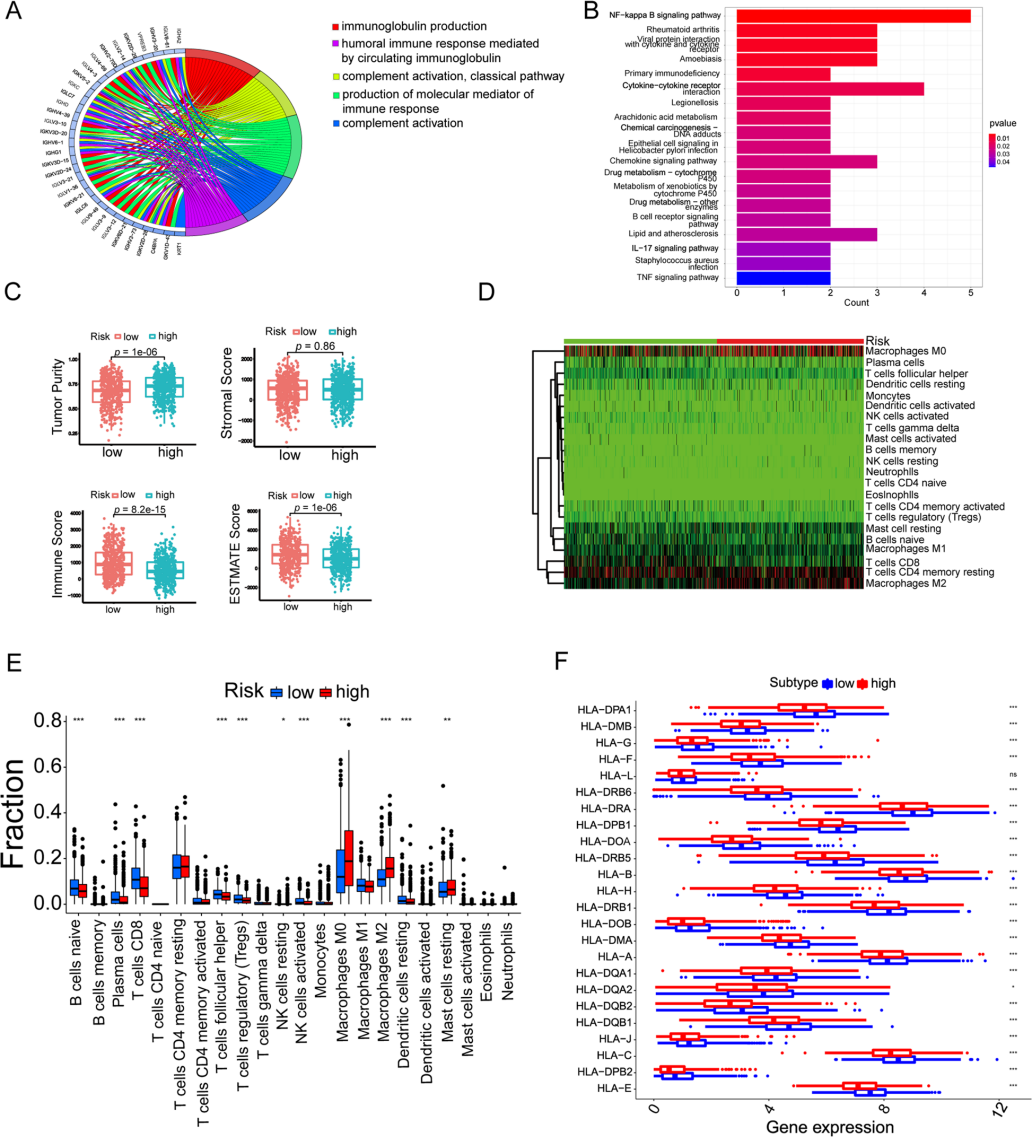
Relative proportion of immune infiltration in high-risk and low-risk groups. **A** GO analysis of DEGs. **B** KEGG pathway analysis of DEGs. **C** The immune microenvironment among the two risk groups. **D** Heatmap of immune cell differences between two subgroups. **E** Immune cells the between two subgroups. **F** Gene expression of HLA gene sets between two distinct clusters

To elucidate potential pathways for gene enrichment in high- and low- risk groups, we analyzed 23 up-regulated and 97 down-regulated differential genes (DEGs) between the high-risk group and low-risk group (*p* < 0.05, |logFC|> 1.5, Table S3). GO enrichment analysis indicated that DEGs were enriched in immunoglobulin production, complement activation, classical pathway, production of molecular mediator of complement response, humoral immune response mediated by circulating immunoglobulin in BP, MF, and CC functions (Fig. 5A). In addition, KEGG pathway analysis showed that the IL-17 signaling pathway, cytokine interaction receptor, and viral protein interaction with cytokine and cytokine receptor pathway were the central pathways in the riskScore (Fig. 5B). We postulated that riskScore might play an important role in the tumor immune microenvironment (TIME)**.** ESTIMATE algorithms were used for the immune microenvironment of BRCA. The results showed that ImmuneScore and ESTIMATEScore were lower in the high-risk group while TumorPurity was higher (Fig. 5C, *p* < 0.05). We also observed that naive B cells, Plasma cells, CD8 + T cells, follicular helper T cells, Tregs cells and activated NK cells were higher in the low-risk group, while M0, M2 macrophages and resting Mast cells were higher in the high-risk group (Fig. 5D). Heatmap indicated the distribution of immune cells in high- and low- risk groups (Fig. 5E). Further, we investigated the relationship between HLA genomes and risk scores. The results showed that the expressions of HLA genomes were markedly higher in the low-risk group (Fig. 5F). These data suggested that immune response was more active in the low-risk group.

### The negative correlation between riskScore and immune checkpoint expression

**Fig. 6:**
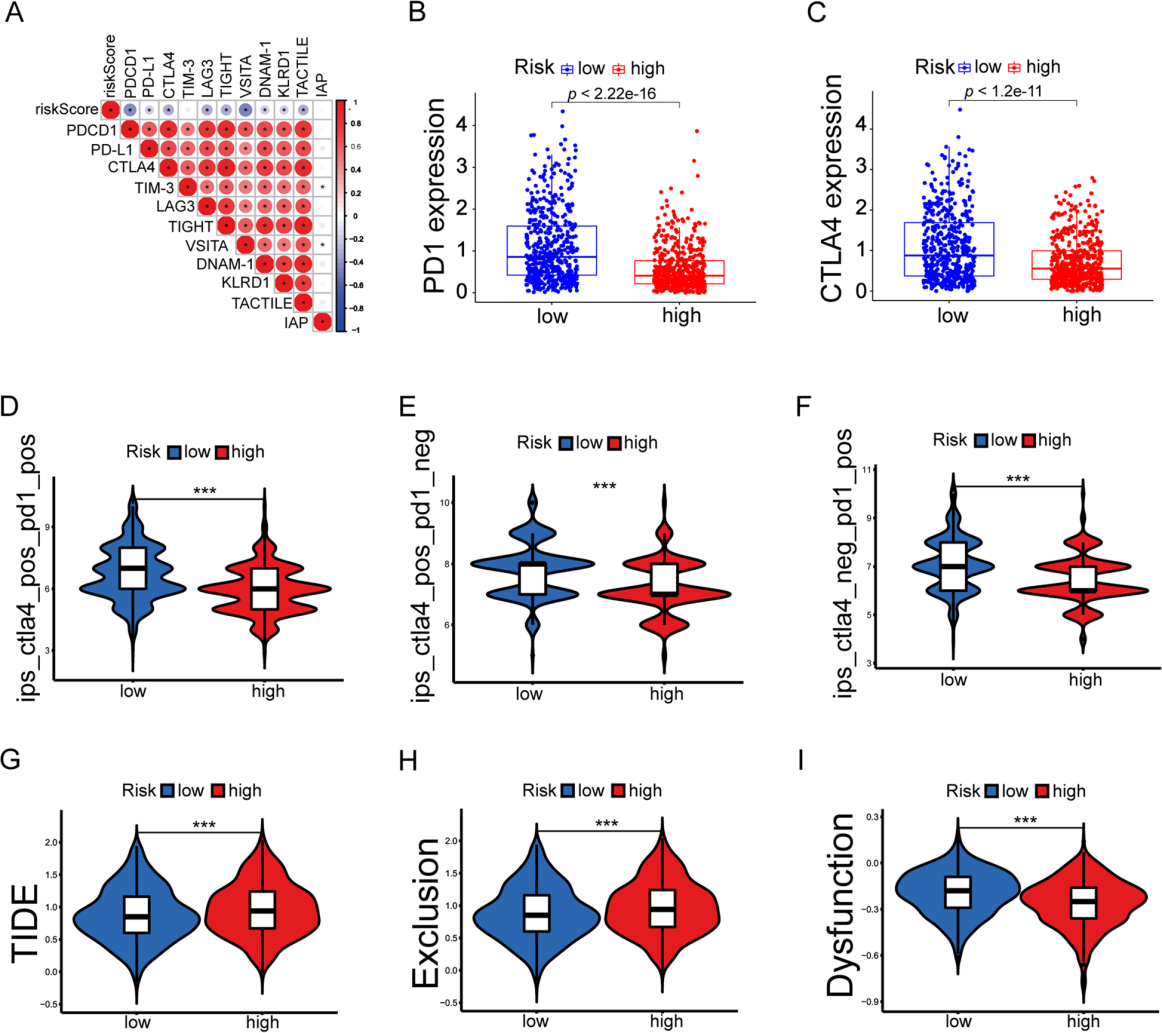
The estimation of prognosis model in immunotherapy response. **A** Correlation of riskScore with immune checkpoints. **B** PD1 expression in high and low risk groups. **C** CTLA4 expression in high and low risk groups. **D**-**F** PD1 and CTLA4 immunotherapy in TCIA. **E** CTLA4 immunotherapy in TCIA. **F** PD1 immunotherapy in TCIA. **G** Relationship between high and low risk groups and TIDE scores. **H** Relationship between high and low risk groups and exclusion. **I** Relationship between high and low risk groups and Dysfunction

To further investigate the variation between prognostic models and immunotherapies, we first assessed the correlation between eleven immune checkpoint molecules and riskScore. We found that immune checkpoint molecules were negatively correlated with risk scores (Fig. 6A). Following this, we looked at the differences in their expression in the high and low risk groups, with the commonly used immune checkpoints PD1 and CTLA4 both highly expressed in the low-risk group (Fig. 6B-C, *p* < 0.05), and all other immune checkpoint molecules, except TIM-3 and IAP, which were not statistically significant, highly expressed in the low-risk group (Figure S9, *p* < 0.05). Additionally, we validated the above immune checkpoints in the GSE6130 dataset (Figure S10). Further, we validated the immune efficacy of the prognostic models via two publicly available data (TCIA and TIDE). The results showed that immunotherapy had better effectiveness in the low-risk group (Fig. 6D-I). Taken together, these data demonstrated that the low-risk group was more sensitive to ICB (immune-checkpoint blockade) treatment than the high-risk group.

### Chemotherapy agents and small molecule targeted drugs for breast cancer patients with high-risk LMRGs

**Fig. 7:**
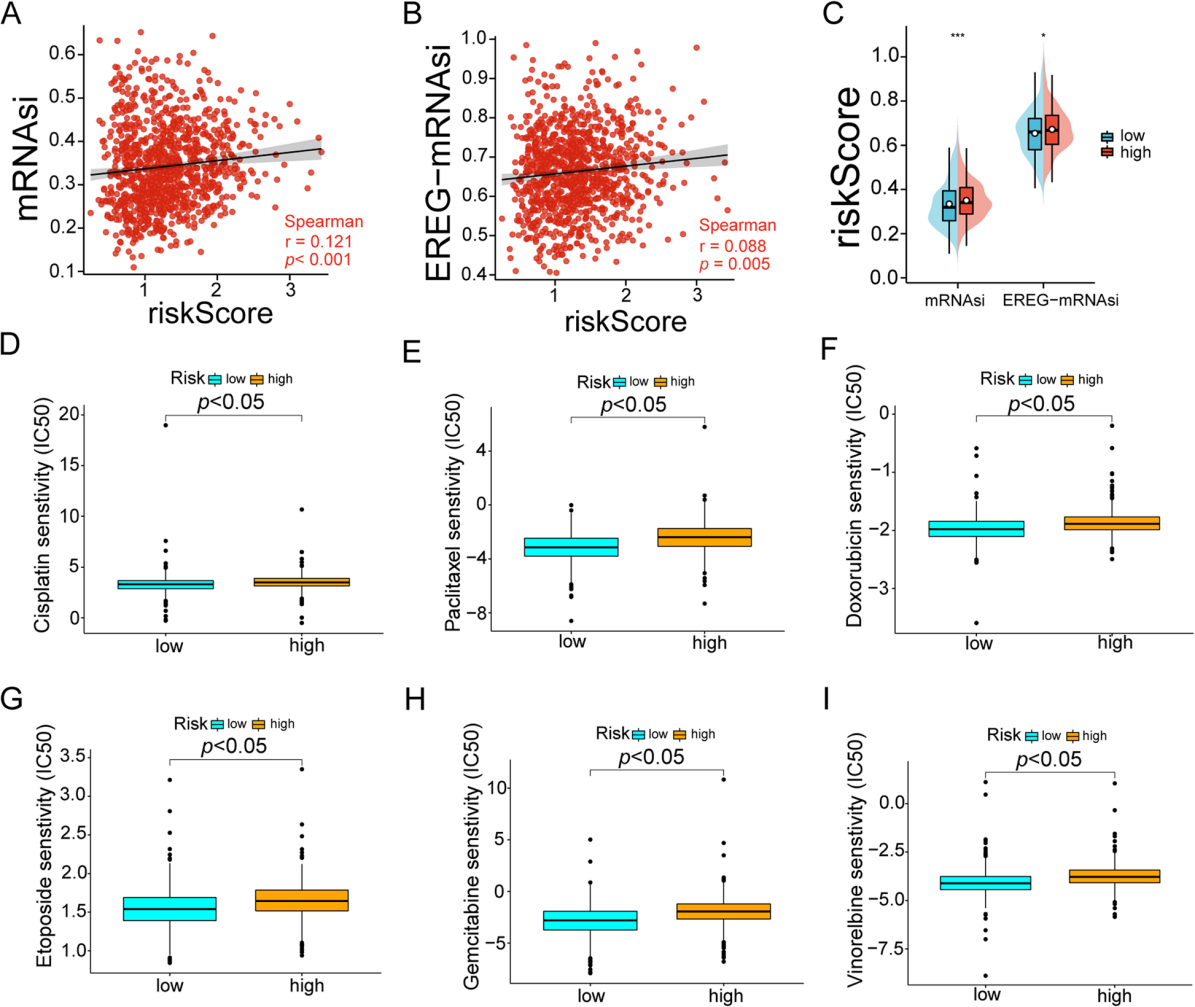
The tumor stemness index of breast cancer reflects its response to chemotherapy. **A** and **B** Relationship between TSI and risk score. **C** Pod plot showing the relationship between risk scores and TSI (*: *p* < 0.05;**: *p* < 0.01;***: *p* < 0.001). **D**-**I** The chemotherapy response of two prognostic subtypes for six common chemotherapy drugs ((**D**) Cisplatin; (**E**) Paclitaxel; (**F**) Doxorubicin; (**G**) Gemcitabine; (**H**) Etoposide and (**I**) Vinorelbine)

Previous studies have shown that LMRGs were association with chemoresistance through the activation of tumor stem cells. Therefore, we addressed the relationship between tumor stemness index (TSI) and riskScore in BRCA. The results revealed that both mRNAsi and epigenetically regulated mRNAsi (EREG-mRNAsi) were positively correlated with riskScore (*r* = 0.088, *p* = 0.005; *r* = 0.121, *p* < 0.001, respectively, Fig. 7A-B). The pod plots showed that both mRNAsi and EREG-mRNAsi were higher in the high-risk group (Fig. 7C, *p* < 0.05, *p* < 0.001). Further, we performed IC50 to estimate cisplatin, paclitaxel, doxorubicin, gemcitabine, etoposide and vinorelbine (Fig. 7D-I). The data showed that compared with the low-rated group, the IC50 of cisplatin, paclitaxel, doxorubicin, gemcitabine, etoposide and vinorelbine in the high-risk groups were increased. These data indicated that riskScore might serve as an indicator for chemotherapy.

Further, the CMap database (https://portals.broadinstitute.org/cmap/) was used to screen for small molecule drugs. The eight small molecule drugs were screened based on DEGs in high- and low- risk groups (*p* < 0.05, enrichment < 0, Table S4). 2D structural imagings of tolnaftate, rifampicin, clenbuterol, anisomycin, fusaric acid, withaferin A, spironolactone, MG-262, desipramine were displayed in PubChem (Figure S11).

## Discussion

In view of the highly variable prognosis of BRCA, it is crucial to establish a robust categorizer to stratify patients with different risks and prognoses. It is essential to maximize the benefits from personalized treatment and timely follow-up. There have been invested in exploring the complex mechanisms of BRCA. However, it remains far from satisfactory via understanding TME, treatment targets and prognostic factors. In this study, we constructed predictive models of twelve LMRGs and comprehensively explored their transcriptomes, gene mutations, copy number variants, immune microenvironment and tumor stemness, which intended to construct a novel strategy to solve important clinical question.

We found that LMRGs screened by LASSO regression analysis were associated with survival in BRCA patients in the training, test, and external validation sets. Surprisingly, the trained model showed that 5-year survival prediction was higher accuracy on the training set compared to the results for 1-year survival prediction. The same results were also shown in the test set. Furthermore, the inverse effect of LMRGs was significantly associated with prognosis, even after stratifying patients by clinicopathological risk factors. And the LMRGs showed different values of risk scores in the context of different molecular subtypes in breast cancer. For instance, the relationship between prognosis and the subgroups (Luminal A and TNBC) was associated, which has been reported previously [17–19]. Further, we found that the riskScore could be an independent prognostic factor for BRCA using univariate and multivariate Cox regression analysis. The hazard ratio of riskScore was higher than the stage, which means a better prognostic value. Through combining risk score with age, stage and ER, PR, HER2 receptor, we constructed a prognostic model of BRCA using nomogram and validated its accuracy.

Previous studies only have examined the effects of LMRGs at the transcriptome level and single-nucleotide mutations and copy number variants have not been thoroughly studied in breast cancer [20]. This study systematically analyzed the interaction and prognostic network of the twelve LMRGs. And in this study, it performed a comprehensive analysis of their mutations and copy number variants which could provide a more thorough understanding of the LMRGs.

Lipid metabolic reprogramming played a vital role in the tumorigenesis and progression of BRCA [21, 22]. It has been reported that the ability of lipid metabolism in tumor was an essential mechanism to evade immune surveillance [23, 24]. Cancer cells require large amounts of energy to undergo division, and conventional glycolysis can no longer satisfy its energy requirements [25, 26]. Hence, cancer cells resort to lipid metabolism to provide their needs for growth. In this process, lipid metabolism inhibitd the release of chemokines which affected the recruitment of immune cells to cancer cells [27–29]. It is well reflected in our GO and KEGG analysis in the high- and low- risk groups. Interestingly, tumor purity was higher in the high-risk group, and immune and stromal components were higher in the low-risk group. The data suggested that tumor tissues with a higher proportion of tumor cells had a more remarkable ability to reprogram lipometabolism, which inhibited the immune microenvironment in some extent [30–32]. In addition, B cells and T cells were lower in the high-risk group, confirming that lipometabolism can cause immune cell depletion. Interestingly, M2-type macrophages were more numerous in the high-risk group, and it has been shown that tumor metabolic reprogramming induced the transformation of macrophages from M1-type to M2-type [33].

Previous studies show a relationship between breast cancer stem cells and chemotherapy resistance [34]. In our research, low-risk patients had a low stemness index and were more sensitive to chemotherapeutic agents, which suggested twelve genomes were as targets for therapeutic intervention. There was different in the immune checkpoint between the high- and low-risk group and it’s better to immunotherapy response in low-risk group. This data suggested that risk scores can influence the outcome of immunotherapy and prognostic models can determine which types of patients are more likely to respond to immunotherapy in BRCA. With the popularization of DNA sequencing technology, we have entered the era of precision medicine. They found the abnormality of gene expression and saw the difference between each patient and therapeutic efficacy [35, 36]. Immunotherapy has become a problem that every clinical need to solve. Interestingly, lipids are an important fuel source for energy production and most of signaling pathways and enzymes are affected in cancer cells, which means lipid metabolism is a central role in cancer biology [9]. According to current concepts, cancer is mainly driven by oncogenes to promote unlimited growth and metastasis. It usually involves the constitutive activation of growth factor receptors and downstream signaling. Still, it also consists of reprogramming metabolic processes to provide substrates and energy for cancer cells in the changing microenvironment [37].

We predicted these potential targets using riskScore and constructed from twelve lipometabolism genomes. It enabled chemotherapy and immunotherapy to be used in patients who were not sensitive to drugs. However, there are still some challenges to translating these targets into clinical therapeutics. In particular, the molecular types of BRCA has not been included in subsequent treatment. Secondly, how the twelve genes cause the tumorigenesis and development of BRCA and the related mechanisms still need further verification using in vivo and in vitro experiments.

In summary, this study comprehensively evaluated the role of LMRGs in the prognosis and immune microenvironment and explored the molecular mechanisms in BRCA. The LMRG-based risk model was successfully predicted the overall survival of patients and pointed out the tumor immune microenvironment. In addition, our results showed that the characteristics of tumor stemness could affect the chemotherapeutic efficacy and immune-related signaling pathways might mediate the function of LMRGs in BRCA. Our work provided an innovative perspective for future research and targeted therapies. Further studies were required to verify the prognostic value of LMRG-based risk model and its potential mechanisms. In a word, it’s important for the riskScore to serve as a preventive or therapeutic strategy in BRCA.

## Supplementary Information

**Additional file 1. **

**Additional file 2. **

## Data Availability

Publicly available datasets were used in this study. These data can be found in The Cancer Genome Atlas (TCGA) database (https://portal.gdc.cancer.gov/) and GEO database (https://www.ncbi.nlm.nih.gov/geo/). These databases are public databases, and their websites were provided in the manuscript.
