# Supplementary material for: Novel lipometabolism biomarker for chemotherapy and immunotherapy response in breast cancer
Source: BMC Cancer. 2022 Oct 1;22:1030. doi: 10.1186/s12885-022-10110-8 (PMC9526348; doi:10.1186/s12885-022-10110-8)
Supplement: Supplementary file 1 — Additional file 1. [file 12885_2022_10110_MOESM1_ESM.docx]

**Supplementary figures and legends**

**
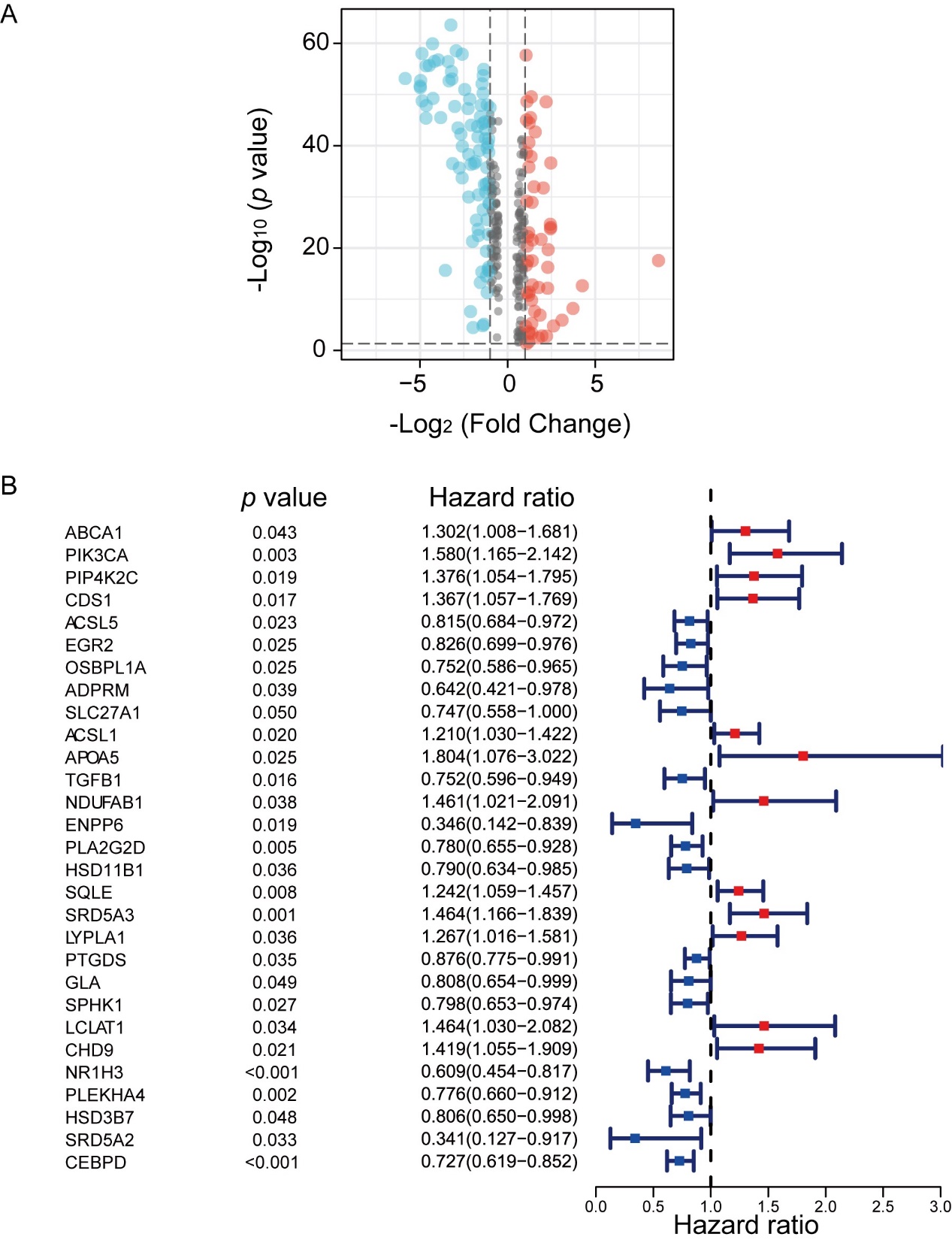
**

**Figure S1.** Differential gene expression of lipometabolism-related genes in breast cancer. (A) Differential genes of LMRGs in tumor tissues and normal tissues. (B) Univariate Cox regression analysis of differential genes.


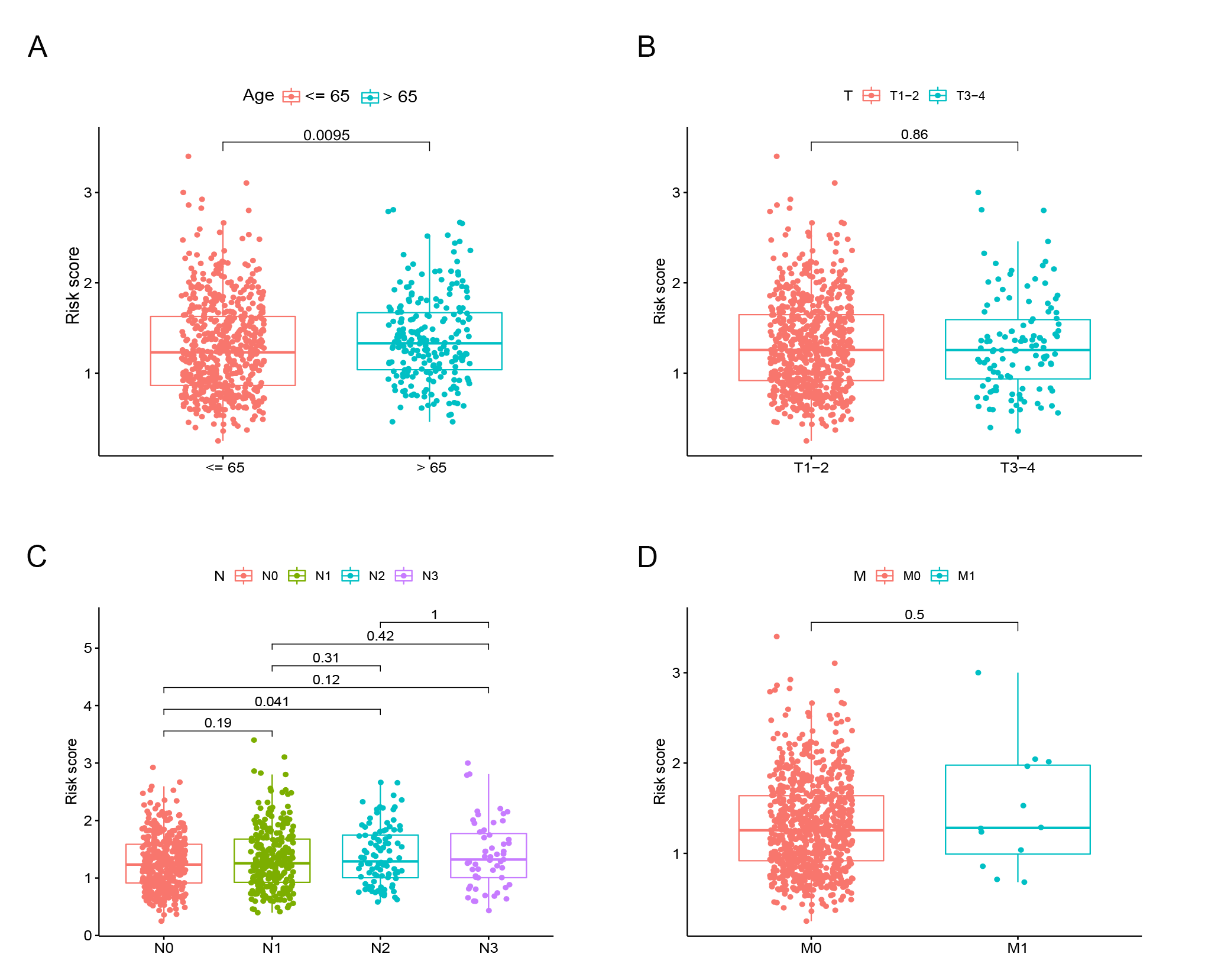
**Figure S2.** The correlation of riskScore with patients’ clinicopathological characteristics. (A) Association between age and riskScore. (B) Association between T stage and riskScore. (C) Association between N stage and riskScore. (D) Association between M stage and riskScore.


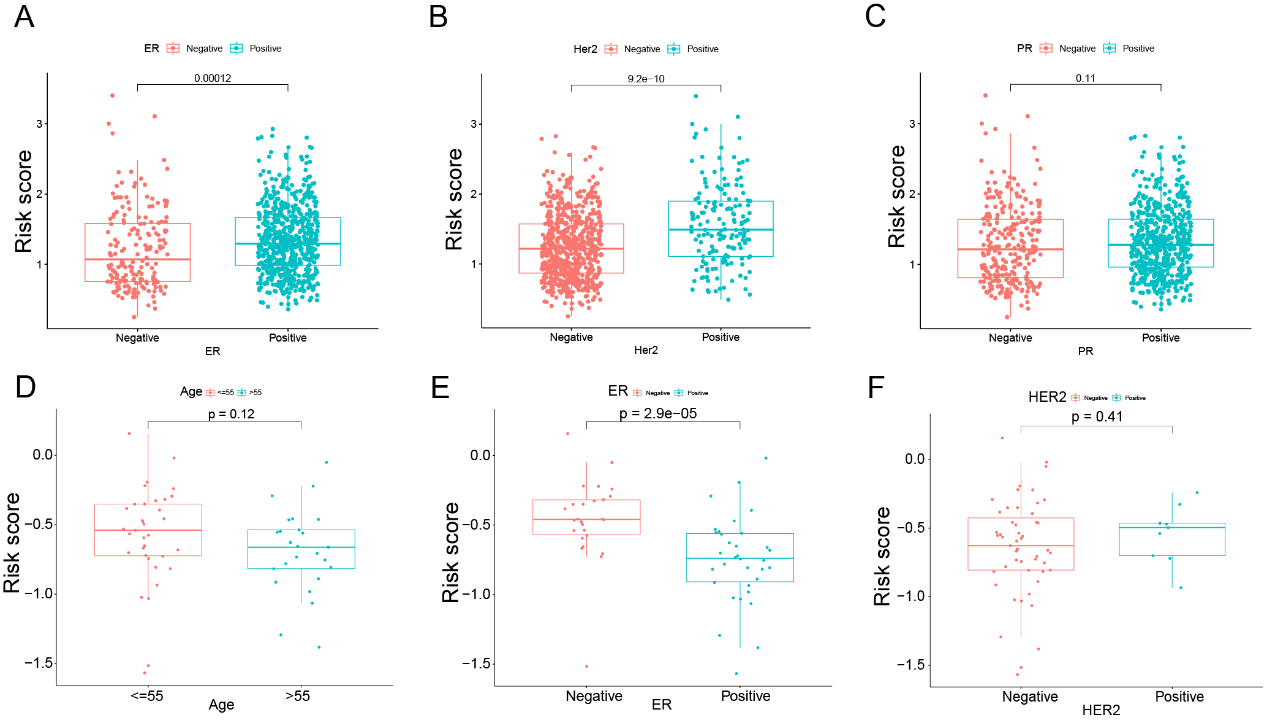


**Figure S3.** The correlation of riskScore with patients’ clinicopathological characteristics. (A) Association between Her2 receptor and riskScore. (B) Association between ER receptor and riskScore. (C) Association between PR receptor and riskScore. (D) Association between Age and riskScore based on GSE6130. (E) Association between ER receptor and riskScore based on GSE6130. (F) Association between HER2 receptor and riskScore based on GSE6130.

**
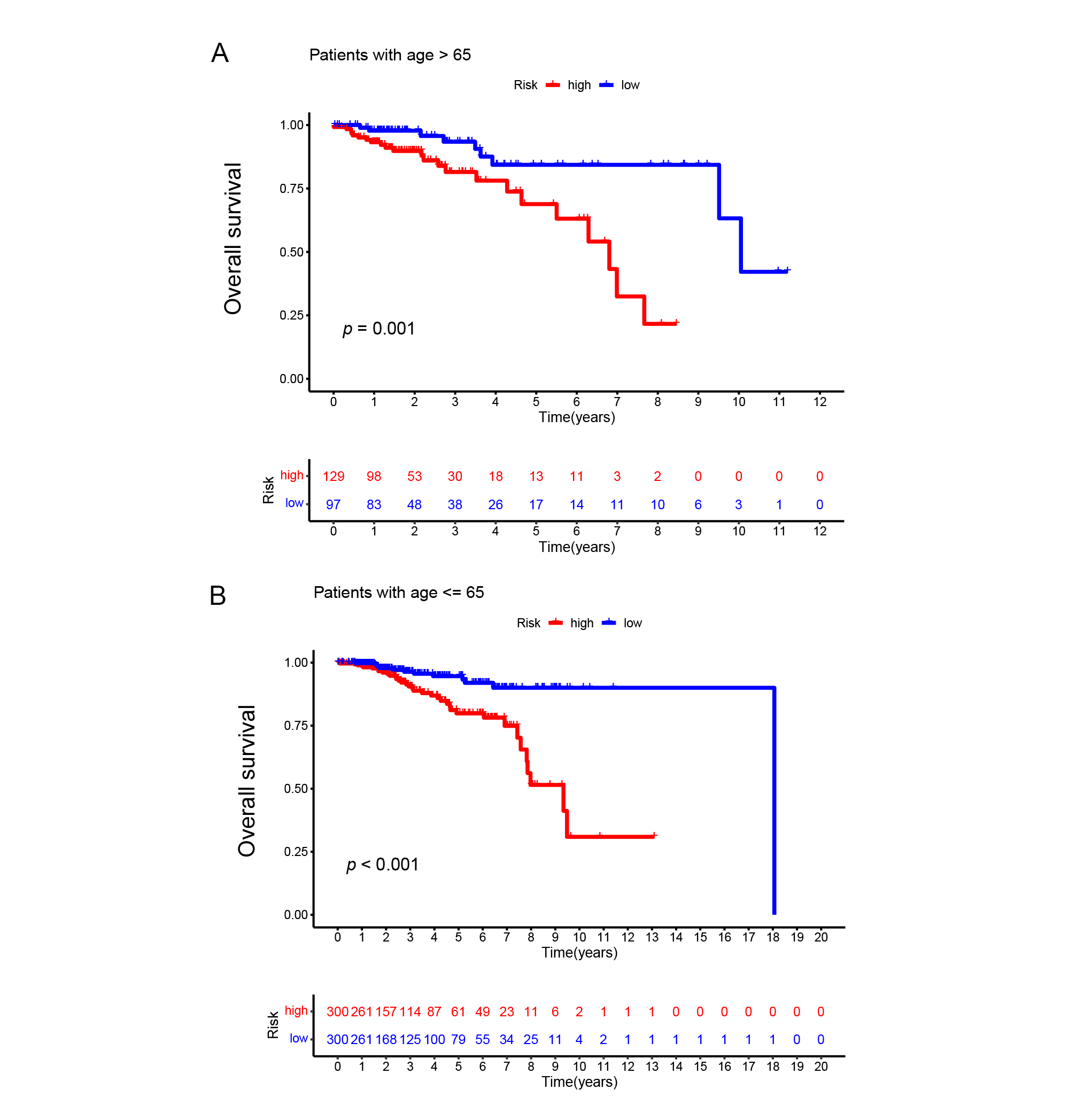
Figure S4.** (A and B) Survival rates of age in high and low risk groups.

**
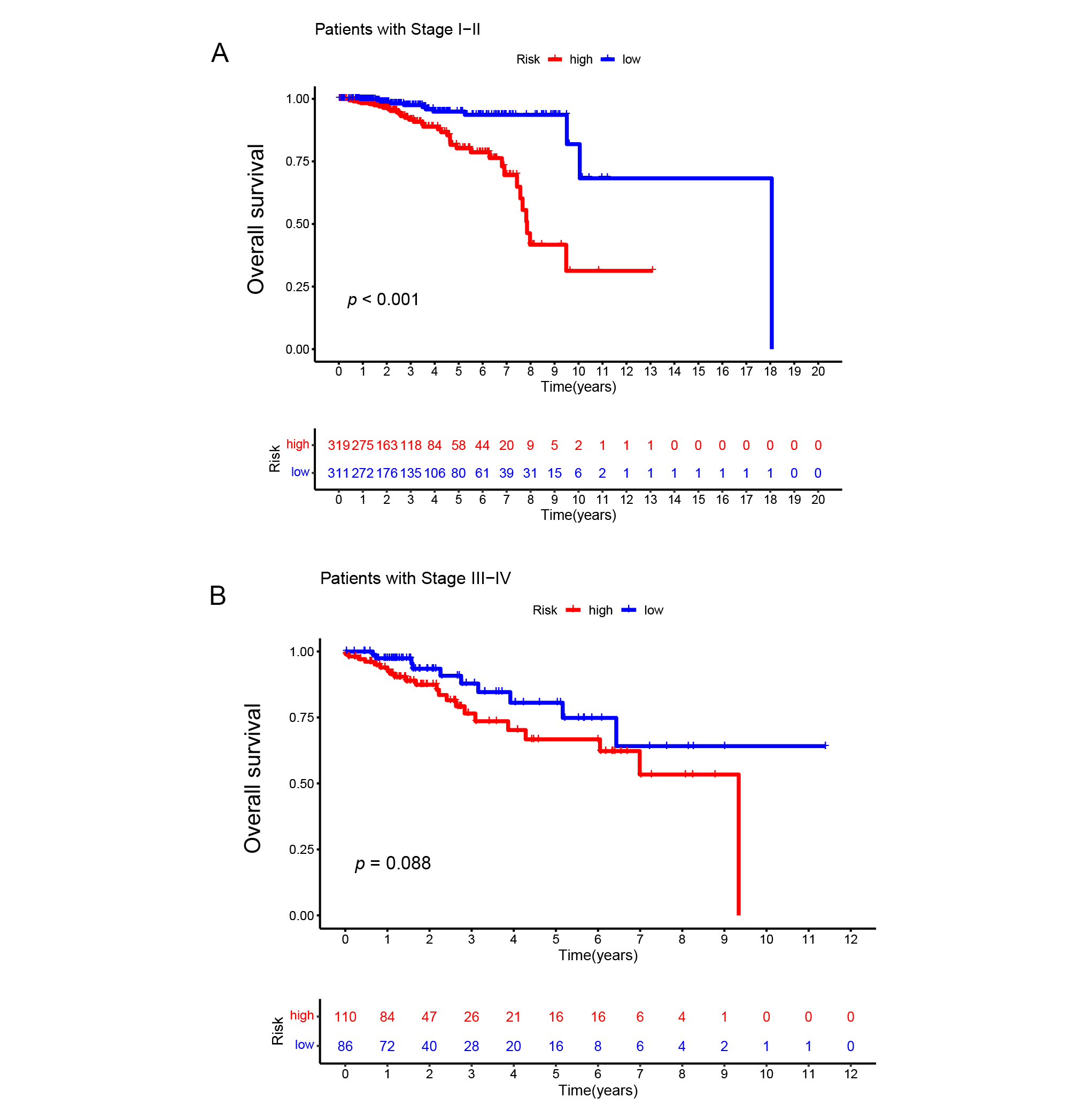
Figure S5.** Survival rates of stage in high and low risk groups. (A) Stage I-II. (B) Stage III-IV.


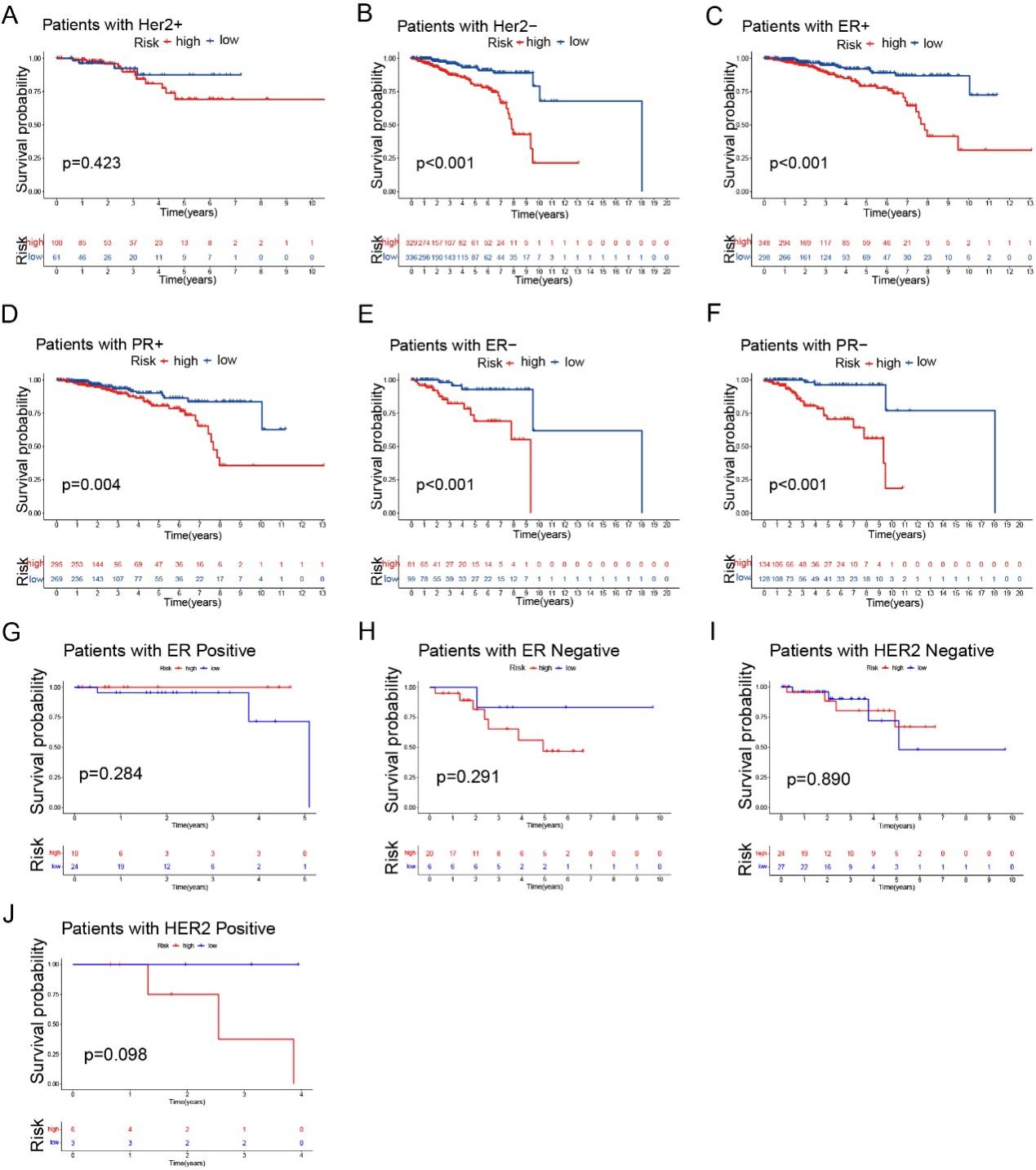
**Figure S6.** (A-F) Survival rates of Her2, ER, PR in high and low risk groups based TCGA. (G-J) Survival rates of ER, Her2 in high and low risk groups based on GSE6130.


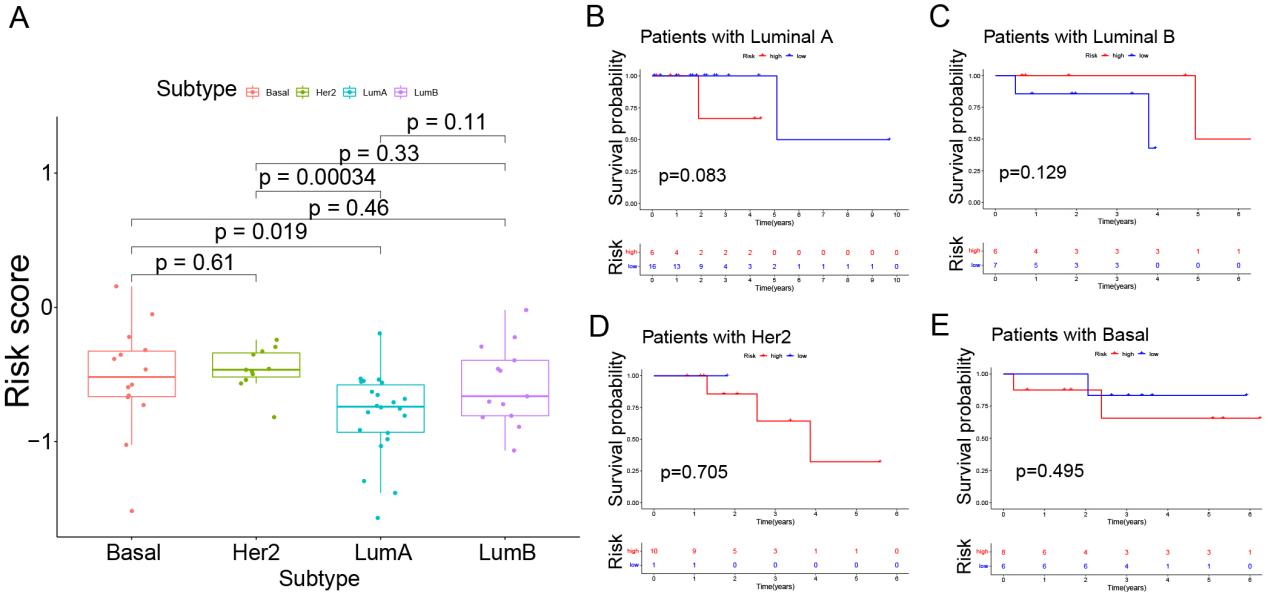


**Figure S7.** (A) Association between risk scores in LMRGs and the four molecular types of breast cancer based on GSE6130. (B-E) KM survival curves for breast cancer molecular type in high-and low-risk groups based on GSE6130. (B) Luminal A; (C) Luminal B; (C) Her2; (D) Basal like.


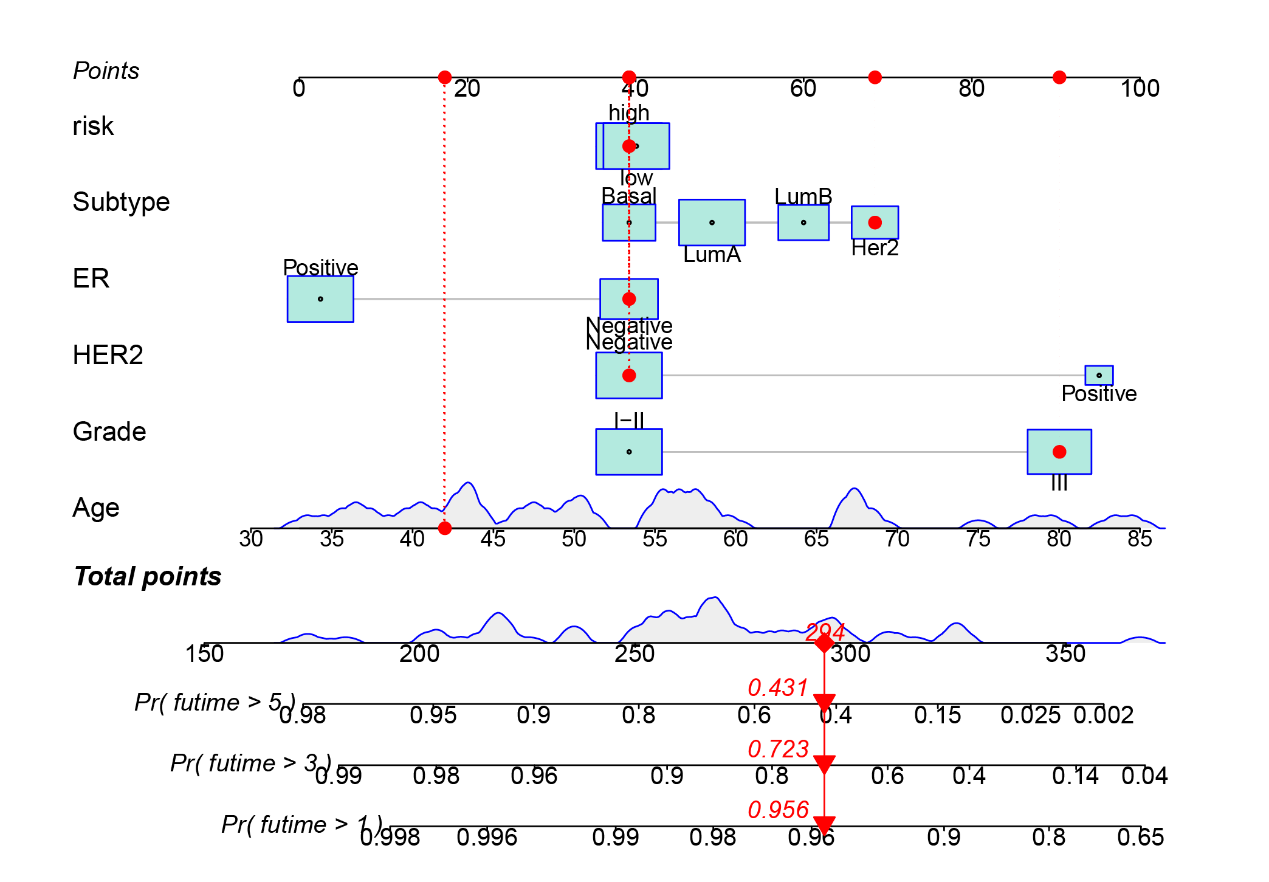


**Figure S8.** Nomogram for predicting the probability of patient death at 1, 3 or 5 years OS based on the risk score constructed by GSE6130.

**
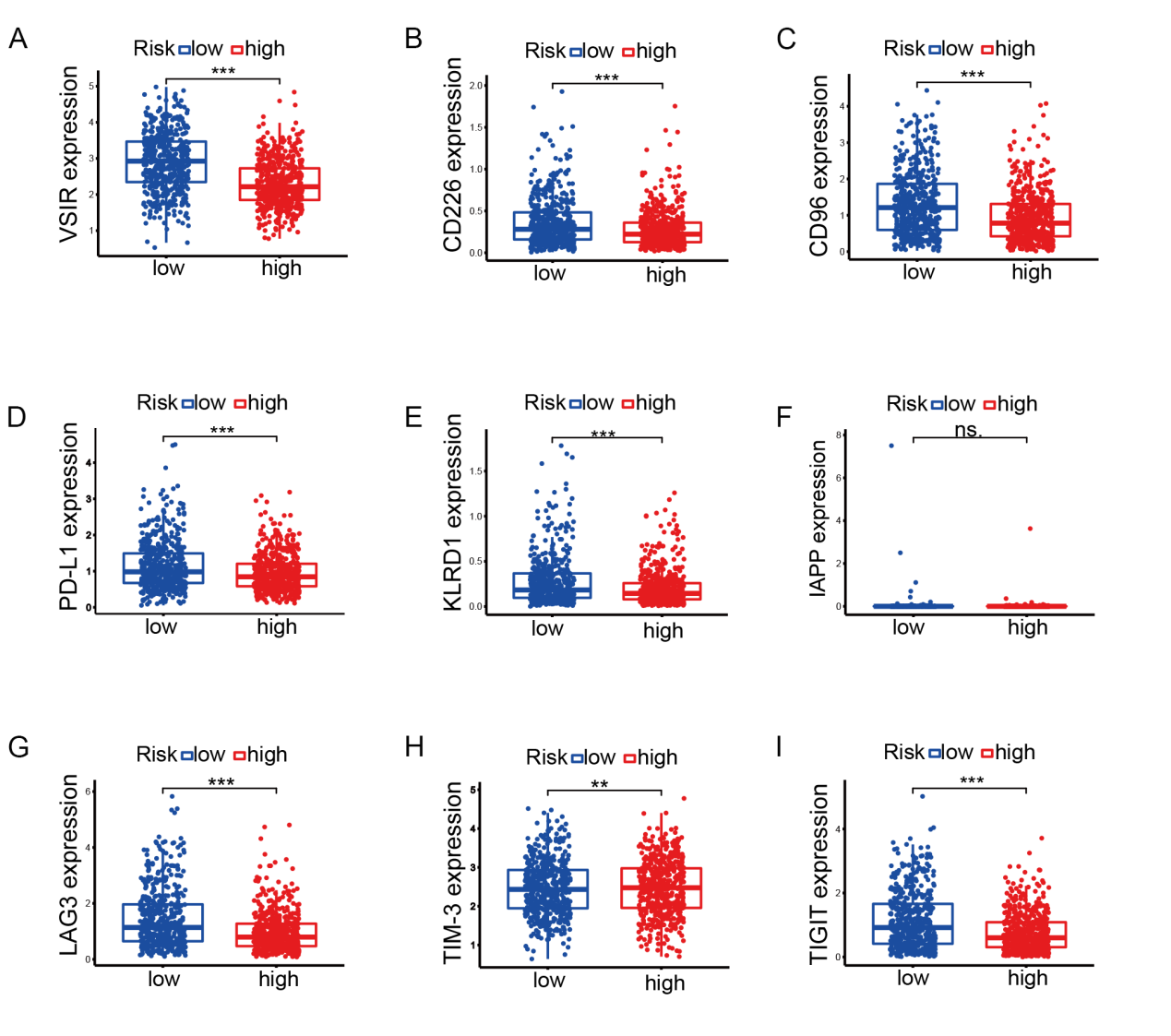
****Figure S9.** Immune checkpoints in relation to high and low risk groups. (A) VSIR. (B) CD226. (C) CD96. (D) PD-L1. (E) KLRD1. (F) IAPP. (G) LAG3. (H) TIM-3. (I) TIGIT.


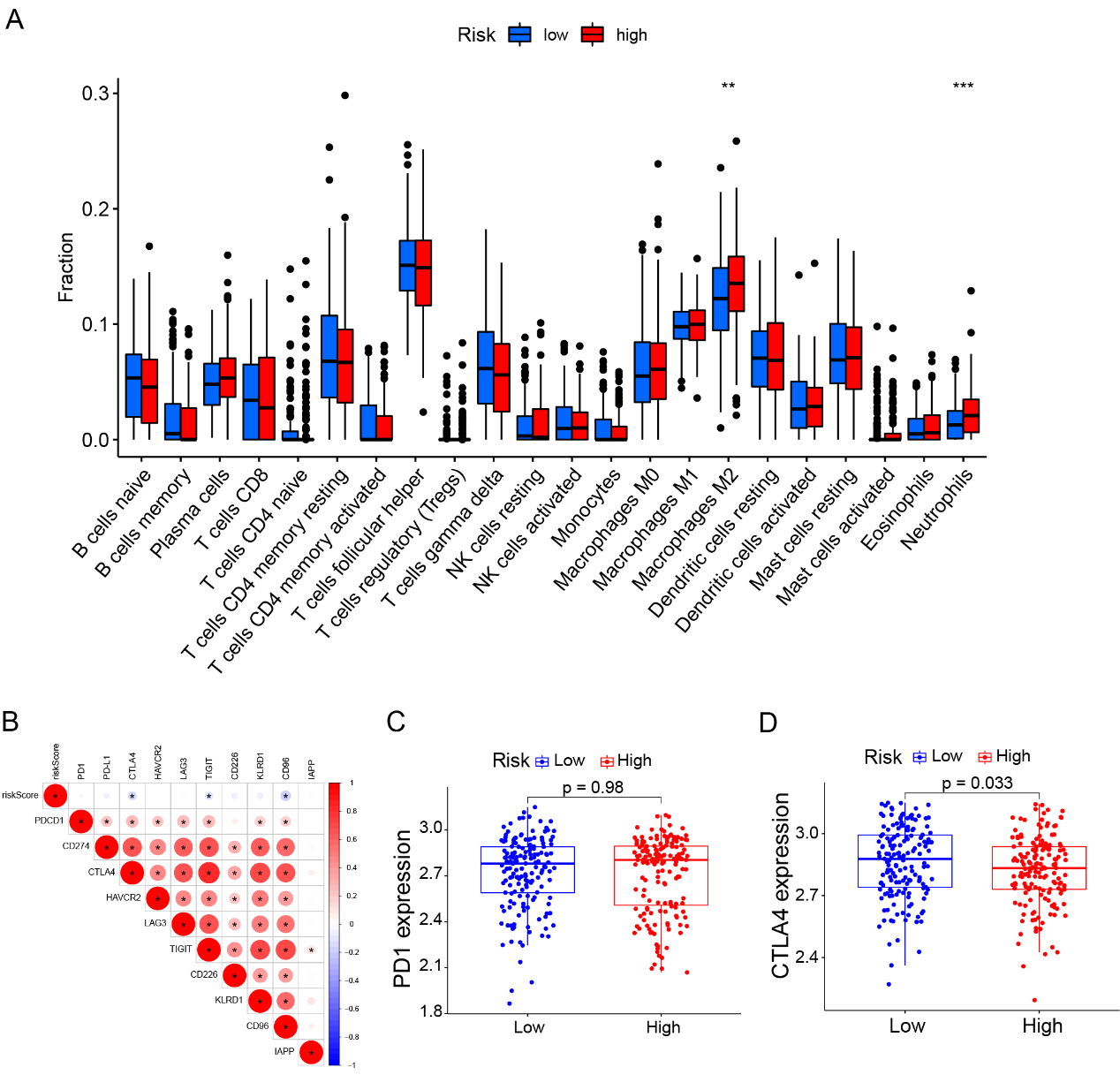


**Figure S10.** Immune cell infiltration and immune checkpoint expression based on the GSE6130 constructed risk cohort. (A) Differential infiltration abundance of immune cells in high- and low-risk cohorts. (B) Correlation of riskScore with immune checkpoints. (C) PD1 expression in high and low risk groups. (D) CTLA4 expression in high and low risk groups.


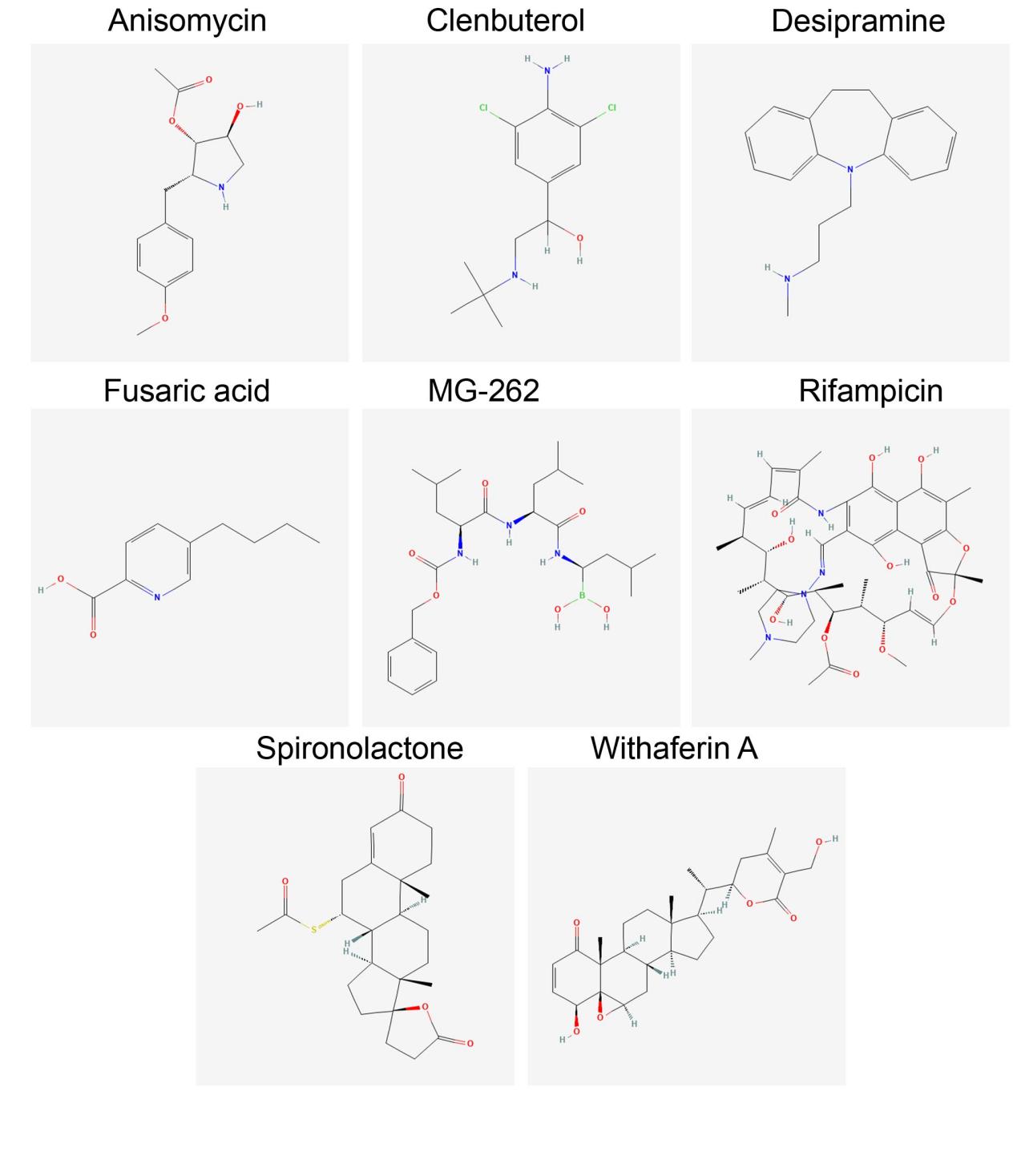


**Figure S11.** Estimation of potential therapeutic agents for breast cancer based on LMRGs.
