# Supplementary material for: Novel lipometabolism biomarker for chemotherapy and immunotherapy response in breast cancer
Source: BMC Cancer. 2022 Oct 1;22:1030. doi: 10.1186/s12885-022-10110-8 (PMC9526348; doi:10.1186/s12885-022-10110-8)
Supplement: Supplementary file 2 — Additional file 2. [file 12885_2022_10110_MOESM2_ESM.docx]

**Supplementary tables**

**Supplementary table 1 Lipometabolism-related genes in breast cancer**

| MOGAT2 | SLC44A5 | ABCB4 | AKR1C1 | PCYT1A | SPHK2 | CROT | PLA2G1B | NR1H2 |
| --- | --- | --- | --- | --- | --- | --- | --- | --- |
| SIN3B | SBF1 | CYP7B1 | SPTSSA | PTGES2 | CYP4F8 | UGCG | HSD17B8 | PPARD |
| CTSA | GLIPR1 | SLC27A3 | ARSE | CRAT | TBL1X | GDE1 | FITM2 | CYP4F3 |
| MED8 | GK | RGL1 | DGKQ | GLB1 | ORMDL1 | PSAP | SPTLC3 | SLC44A4 |
| DGKD | LSS | PEMT | HMGCS2 | PECR | CEPT1 | SLC22A5 | PRKACG | MED21 |
| WNT1 | TNFRSF21 | PI4KA | KLF4 | NSDHL | PLA2G2F | PCTP | BCHE | GNPAT |
| ACOX2 | HACL1 | FABP4 | MORC2 | CYP21A2 | AACS | ACLY | SGPP2 | OCRL |
| CYP2E1 | HSD3B7 | SUMO2 | ARNTL | NCOR1 | HSD11B2 | GLTP | SP1 | CYP51A1 |
| STARD5 | HAO2 | TGS1 | DGKE | ACOX1 | LPCAT2 | STAR | AKR1C4 | PLEKHA3 |
| CYP2D6 | ACOT9 | DGKI | INPP4A | TXNRD1 | PRKD1 | CYP3A4 | PGS1 | DDHD1 |
| GALC | STARD10 | SGPP1 | FABP5 | CYP4A22 | CSNK2A1 | CLOCK | CYP11B2 | ACADM |
| MED31 | RUFY1 | RAB4A | LIPE | UGT1A9 | DPEP3 | PLD3 | SPTSSB | PIK3R2 |
| CDK8 | CYP4A11 | CYP39A1 | FABP7 | PLA2G6 | PIK3CA | EP300 | BAAT | EGR2 |
| LPCAT3 | BDH2 | MTF1 | CHAT | CEBPD | CGA | CDK4 | PITPNM2 | AGPAT5 |
| PRKACB | AKR1C3 | PLA2G4C | PON3 | INPPL1 | SLC2A4 | PIP4K2C | ETNK1 | PPARGC1A |
| AKR1C2 | CCNC | MTMR7 | LPIN2 | PLA2G15 | ANGPTL4 | CYP2U1 | STARD4 | PLEKHA6 |
| ABCA1 | LHB | PLA2G4A | ESYT1 | NCOR2 | FABP3 | SLCO1B3 | TBXAS1 | PLA2G2C |
| ANKRD1 | SC5D | ACOX3 | PIK3R3 | FDPS | MVK | TNFAIP8L3 | ELOVL3 | DHCR24 |
| PON1 | SREBF2 | PPM1L | MBTPS1 | MCEE | EPHX2 | SEC24B | HADH | SACM1L |
| VAC14 | GBA | TMEM86B | VDR | LTA4H | OSBPL2 | MED16 | PIP5K1A | ZNF467 |
| PTGS2 | ACOT8 | AKR1D1 | GPCPD1 | ACER2 | MECR | FAR2 | FDX1 | MED13L |
| SYNJ1 | ARSK | PLD6 | MED26 | ELOVL6 | CHKB | ORMDL2 | DGKZ | PNPLA2 |
| OSBPL5 | CIDEC | DHCR7 | PCK1 | CYP4F2 | PLA2G4F | ACAT1 | B4GALNT1 | PRKD3 |
| MBOAT1 | SRD5A2 | PLA2G10 | ABHD5 | FASN | FAR1 | GPAM | PCCA | PRKAB2 |
| PPARA | TSPO | GGT5 | CDS2 | ESRRA | PMVK | MTMR12 | PPP1CA | CYP2C9 |
| GC | SAMD8 | CYP11A1 | ABCC1 | AGPAT2 | PLEKHA1 | DHRS7B | ARSG | SLCO1B1 |
| PLIN1 | ELOVL5 | GRHL1 | POMC | OSBPL10 | NFYA | CSNK2B | PLD4 | ACSF2 |
| MED1 | CDIPT | ALDH3A2 | JMJD7-PLA2G4B | PITPNM1 | AWAT1 | INSIG1 | ARF1 | INPP5J |
| MED28 | SGMS2 | PLA1A | RAN | ACHE | NR1H3 | CUBN | ACACB | TPTE |
| BDH1 | PI4KB | CYP1A2 | PPT2 | ARSF | HSD17B13 | FAAH | GPX1 | PIK3C2B |
| CPT2 | ASAH1 | INPP5E | AKR1B15 | MED24 | FA2H | GPX2 | GGT1 | GSTM4 |
| PI4K2A | DGAT2L6 | LCAT | CYP8B1 | HPGD | CBR4 | TNFAIP8 | LPL | THEM4 |
| PTGR1 | AGMO | NFYB | RXRA | KPNB1 | PLIN2 | PLA2G3 | HDAC3 | MGLL |
| RXRB | G0S2 | LPCAT4 | ARSJ | DGKH | ELOVL7 | CDS1 | ORMDL3 | PTGIS |
| THEM5 | NCOA6 | ACSBG1 | HTD2 | PRKACA | MTMR14 | PIK3C3 | PCCB | ACSS3 |
| LPIN1 | RELA | ARSA | ALPI | LYPLA2 | ELOVL2 | CERS4 | ACSL5 | ACOT13 |
| FIG4 | DDHD2 | NR1D1 | LIPI | PPARG | SMPD2 | ECI1 | CYP46A1 | PCYT1B |
| STARD3 | PNPLA8 | KLF5 | SLC25A20 | NRF1 | STARD7 | PLA2G4E | LCLAT1 | SPTLC2 |
| ALB | CYP17A1 | CYP2R1 | DEGS1 | MED19 | TBL1XR1 | MED18 | PTPN13 | IDI2 |
| AGPAT3 | NEU1 | INPP5D | PLEKHA2 | ALOX15B | LGMN | MTMR10 | LPGAT1 | DBI |
| PNPLA4 | MED15 | PTGS1 | PPP1CC | INSIG2 | PIK3C2G | ACOT4 | MED20 | MED29 |
| SPTLC1 | CPT1B | NUDT19 | PIK3R5 | DECR1 | CPNE3 | MMAA | LBR | SYNJ2 |
| MED27 | MLYCD | PLA2G4D | PON2 | GPAT2 | SMPD1 | SLC44A3 | PNPLA7 | CHKA |
| PLA2G5 | HMGCS1 | ADIRF | FDFT1 | AMACR | ALAS1 | ME1 | SMPD4 | GK2 |
| MED13 | ACER3 | ABCC3 | PLB1 | ACBD6 | NEU4 | PIP4K2B | OLAH | HEXA |
| MTMR8 | MED25 | LIPH | ALOX12B | NFKB1 | ACSF3 | NEU3 | SGMS1 | CDK19 |
| FABP6 | RORA | HSD11B1 | CPT1A | ABCB11 | ACAA2 | HADHA | SIN3A | ALOX15 |
| UGT8 | MED17 | CYP24A1 | PLIN3 | LYPLA1 | ACOT12 | HSD3B1 | ACP6 | TRIB3 |
| CYP1A1 | TM7SF2 | SGPL1 | ACOT6 | MED23 | ACAT2 | ACADVL | SLC10A1 | TGFB1 |
| EBP | VAPB | ACADS | PLEKHA8 | TNFAIP8L2 | ACAA1 | ALDH3B2 | OXCT1 | ELOVL1 |
| PITPNM3 | CYP4F22 | PIK3CD | GDPD3 | SMPD3 | PLEKHA4 | SEC23A | CERS3 | ARSI |
| HSD3B2 | CD36 | FABP2 | PIP5K1C | SEC24A | ABHD4 | GPD2 | SLC27A1 | PRKD2 |
| SLCO1A2 | THRAP3 | UBE2I | OXCT2 | HSD17B12 | PIP4K2A | VAPA | MED14 | NDUFAB1 |
| FABP12 | PITPNB | PIP5K1B | SRD5A1 | PLD2 | FADS1 | HSD17B3 | PLD1 | ESYT3 |
| LTC4S | ALDH3B1 | ACADL | PISD | PRKAG2 | ALOX12 | MTM1 | MED4 | NR2F2 |
| PLA2G12A | HELZ2 | ABHD3 | MTMR4 | MID1IP1 | NCOA1 | SMARCD3 | B3GALNT1 | ACAD11 |
| CYP1B1 | CPNE1 | IDI1 | HSD17B11 | FABP1 | HADHB | GPD1 | MTMR2 | ACOT11 |
| MBOAT2 | PTEN | MTMR9 | SUMF1 | AHR | OSBPL8 | GDPD5 | TNF | AGK |
| OSBPL7 | CERS6 | ABCD1 | MTMR1 | PLA2G2E | AKR1B1 | PIK3R6 | ETNPPL | PIKFYVE |
| ACOT7 | FHL2 | CSNK2A2 | ARSB | HSD17B4 | PTGDS | MFSD2A | GPX4 | RAB14 |
| ARNT2 | GBA2 | CRLS1 | CERS5 | THRSP | MED12 | NR1H4 | CYP27A1 | CHPT1 |
| SLC44A1 | AWAT2 | ACSL4 | CH25H | PTPMT1 | CEBPB | SRD5A3 | STARD6 | PIK3CB |
| CREBBP | DEGS2 | SUMF2 | ZNF638 | ACBD5 | SCD5 | ETNK2 | PLA2G12B | OSBPL6 |
| PHOSPHO1 | HILPDA | SCAP | HPGDS | AGPAT1 | PNPLA6 | OSBP | KDSR | SBF2 |
| INPP5F | DECR2 | SCP2 | ACSM6 | ALOX5AP | MCAT | SULT2A1 | CYP4F11 | MED9 |
| DGKA | PNPLA5 | CPNE7 | LEP | HMGCL | HEXB | RAB5A | CERS2 | CSNK1G2 |
| TPTE2 | ACSL1 | MBOAT7 | FABP9 | TIAM2 | GLA | SEC24D | DGAT1 | CIDEA |
| MBTPS2 | MTMR6 | CPTP | PLA2G4B | CCND3 | NCOA2 | ALOXE3 | PLEKHA5 | CYP2C8 |
| PPP1CB | PTDSS1 | ECHS1 | GGPS1 | SLC44A2 | ARV1 | ACSL6 | PIK3R1 | PHYH |
| CYP19A1 | FITM1 | ENPP6 | CYP11B1 | INPP4B | CAV1 | CYP4B1 | PRKAA2 | PTGR2 |
| PIAS4 | DPEP1 | PIK3R4 | SLC10A2 | MOGAT3 | NCOA3 | APOA1 | HMGCR | CHD9 |
| OSBPL1A | HSD17B2 | ACOXL | SLC27A5 | NPAS2 | ALOX5 | SQLE | SEC24C | GM2A |
| ADPRM | APOA5 | MED10 | SLC25A17 | LPCAT1 | PNPLA3 | ACBD4 | FDXR | FADS2 |
| FAAH2 | SREBF1 | MVD | PI4K2B | SCD | SAR1B | EHHADH | PPARGC1B | CYP2J2 |
| PIK3CG | CERK | PLA2G2D | ENPP7 | INPP5K | GPD1L | ACER1 | MED7 | MED30 |
| MOGAT1 | ACOT1 | OSBPL3 | APOA2 | MED22 | HMGCLL1 | PLA2G2A | ACACA | ASAH2 |
| ARSH | PEX11A | MSMO1 | PLA2R1 | ACBD7 | CBR1 | ECI2 | LRP2 | FAM120B |
| ARF3 | GPS2 | PCYT2 | ESYT2 | DGKB | WNT10B | PLBD1 | ARNT | SPHK1 |
| PIK3C2A | AGT | ACOT2 | EBF1 | ACAD10 | CYP7A1 | NFYC | ACSBG2 | MAPKAPK2 |
| DGKG | SLC27A2 | ACSM3 | GDPD1 | AGPS | ACSL3 | DPEP2 | HSD17B7 |  |
| PTGES | PPT1 | MTMR3 | OSBPL9 | TECRL | ELOVL4 | GLB1L | TAZ |  |
| CPNE6 | MED6 | SLC25A1 | STS | STARD3NL | DGAT2 | LPIN3 | PTGES3 |  |
| PTDSS2 | TECR | BMX | CYP27B1 | ADIPOQ | HSD17B1 | CYP2C19 | HSD17B14 |  |
| MED11 | ARSD | NEU2 | CERS1 | CARM1 | CEBPA | AGPAT4 | TNFAIP8L1 |  |

There are 751 lipometabolism-related genes in breast cancer

**Supplementary table 2 Differential gene of lipometabolism-related genes in breast cancer**

| gene | conMean | treatMean | logFC | pValue |
| --- | --- | --- | --- | --- |
| CTSA | 17.53433923 | 25.68246939 | 0.550600861 | 7.17E-19 |
| WNT1 | 0.06526196 | 0.045175845 | -0.530690737 | 2.29E-08 |
| AKR1C2 | 18.82679024 | 2.33056029 | -3.014038317 | 2.14E-48 |
| ABCA1 | 9.215390577 | 4.080985028 | -1.175127895 | 2.68E-26 |
| PTGS2 | 4.669382386 | 1.534014097 | -1.605919998 | 8.65E-40 |
| MBOAT1 | 10.90340145 | 16.51780188 | 0.59924344 | 0.002143926 |
| PPARA | 3.249547793 | 1.325634107 | -1.293556339 | 2.23E-45 |
| PLIN1 | 274.2698476 | 8.626982835 | -4.99059613 | 2.33E-52 |
| BDH1 | 2.440683812 | 3.893248441 | 0.673689003 | 2.61E-13 |
| ABHD5 | 11.40940012 | 7.257382613 | -0.652701703 | 3.58E-20 |
| JMJD7-PLA2G4B | 0.775718654 | 0.507800328 | -0.611272168 | 1.84E-12 |
| RAN | 32.73610595 | 52.46617143 | 0.680504798 | 1.47E-35 |
| AKR1C1 | 10.8603012 | 1.071870369 | -3.340861772 | 2.20E-53 |
| DGKQ | 3.089536212 | 4.926789697 | 0.673257608 | 2.99E-18 |
| HMGCS2 | 49.86257567 | 25.8672998 | -0.946827944 | 5.40E-14 |
| KLF4 | 45.83079814 | 8.394815423 | -2.4487469 | 1.08E-51 |
| MORC2 | 7.88012 | 11.14629519 | 0.500274762 | 5.41E-19 |
| FABP5 | 12.87410396 | 7.566384309 | -0.766796064 | 4.25E-19 |
| LIPE | 78.98976977 | 4.127960989 | -4.258164575 | 5.92E-50 |
| ESYT1 | 57.75285811 | 29.90071793 | -0.949712221 | 1.76E-45 |
| PIK3R3 | 10.22932145 | 18.01718048 | 0.816662812 | 9.60E-20 |
| GPCPD1 | 9.178716919 | 6.158793835 | -0.57564466 | 5.57E-23 |
| PCK1 | 11.52662647 | 0.456869598 | -4.657044084 | 4.10E-46 |
| CYP7B1 | 4.471440497 | 2.738164182 | -0.707530724 | 4.86E-23 |
| RGL1 | 13.36382734 | 4.872020134 | -1.455741248 | 8.05E-53 |
| PEMT | 22.11321097 | 11.33941653 | -0.96356212 | 9.84E-21 |
| FABP4 | 1072.006837 | 42.63598349 | -4.65209876 | 2.49E-56 |
| DGKI | 0.513749636 | 0.259986604 | -0.982628179 | 1.70E-23 |
| CYP39A1 | 3.031047877 | 1.332958285 | -1.185185008 | 2.02E-36 |
| PLA2G4A | 6.4355944 | 2.439451498 | -1.399516604 | 5.81E-51 |
| PPM1L | 2.954572203 | 1.485229077 | -0.992263791 | 1.76E-37 |
| TMEM86B | 0.427705063 | 0.633754264 | 0.567307264 | 0.002199985 |
| DHCR7 | 11.15149493 | 24.29004872 | 1.123128258 | 4.12E-21 |
| PLA2G10 | 0.177811378 | 0.610177238 | 1.778880719 | 5.09E-13 |
| ALDH3A2 | 21.57241674 | 14.82887875 | -0.540778294 | 2.92E-23 |
| PLA1A | 1.114537564 | 1.907463667 | 0.775210335 | 0.002600889 |
| CYP1A2 | 0.026951312 | 0.006231748 | -2.112646683 | 2.64E-08 |
| LCAT | 3.163025853 | 1.624118816 | -0.961648168 | 1.19E-36 |
| LPCAT4 | 5.735115081 | 3.908800936 | -0.553096322 | 3.69E-24 |
| SLC2A4 | 8.19766153 | 0.420616347 | -4.284635597 | 1.23E-60 |
| ANGPTL4 | 27.09689352 | 7.66454112 | -1.821856141 | 1.26E-37 |
| FABP3 | 14.0420774 | 9.227804312 | -0.60569707 | 0.002894785 |
| PLA2G4F | 0.41805639 | 1.110142625 | 1.408975576 | 1.58E-13 |
| SPHK2 | 1.748129535 | 2.475854951 | 0.502114704 | 7.94E-12 |
| PLA2G2F | 0.002680287 | 0.016461453 | 2.618632179 | 1.70E-05 |
| HSD11B2 | 2.549607362 | 5.417242809 | 1.087283666 | 5.49E-12 |
| LPCAT2 | 7.672397544 | 3.1759827 | -1.272474421 | 4.07E-42 |
| PRKD1 | 3.319237944 | 1.263130604 | -1.393848237 | 2.12E-54 |
| DPEP3 | 0.079371815 | 0.045514906 | -0.802287693 | 1.58E-16 |
| PIK3CA | 6.64278491 | 4.468076051 | -0.572134459 | 9.24E-27 |
| CGA | 0.029135112 | 11.4048316 | 8.612670586 | 2.82E-18 |
| UGCG | 16.1266549 | 38.05003107 | 1.238450416 | 3.94E-12 |
| CYP3A4 | 0.129839652 | 0.040179673 | -1.692193292 | 1.91E-42 |
| CDK4 | 16.98212626 | 24.90995356 | 0.552705228 | 4.62E-24 |
| PIP4K2C | 15.50778344 | 26.29786649 | 0.761953267 | 3.08E-28 |
| CYP2U1 | 4.365723369 | 1.698077349 | -1.362318541 | 1.22E-55 |
| ORMDL2 | 6.856361495 | 12.42803847 | 0.858083531 | 1.12E-40 |
| ACAT1 | 17.2938624 | 10.47450847 | -0.72337757 | 1.75E-21 |
| GPAM | 55.71385363 | 3.075514536 | -4.179136408 | 3.40E-57 |
| CSNK2B | 13.51928389 | 20.74981198 | 0.618079529 | 2.00E-24 |
| CUBN | 0.506429108 | 0.177188823 | -1.51507273 | 1.27E-48 |
| FAAH | 7.177377874 | 13.57174118 | 0.919077039 | 5.94E-17 |
| GPX2 | 4.330889818 | 2.338225179 | -0.889249596 | 1.55E-20 |
| TNFAIP8 | 3.356645643 | 2.289321773 | -0.552099988 | 2.47E-15 |
| CDS1 | 7.657590708 | 13.50141162 | 0.818147798 | 2.18E-21 |
| SPTSSB | 2.03986148 | 7.947673127 | 1.962061351 | 0.001431226 |
| BAAT | 0.014815936 | 0.034192776 | 1.206541773 | 0.021087449 |
| ELOVL3 | 1.989386018 | 0.62139398 | -1.678743073 | 2.16E-24 |
| HADH | 55.78944568 | 24.94296422 | -1.161359292 | 1.07E-48 |
| PIP5K1A | 11.79359346 | 18.39040433 | 0.640949831 | 4.00E-27 |
| B4GALNT1 | 0.329991846 | 0.769312611 | 1.221139581 | 0.000466327 |
| PCCA | 6.841382829 | 3.436732193 | -0.993250528 | 6.32E-47 |
| PPP1CA | 31.75783342 | 68.15191033 | 1.101641604 | 2.33E-49 |
| ARSG | 2.541104105 | 4.104836009 | 0.691869105 | 9.27E-06 |
| ARF1 | 107.0918757 | 222.4001695 | 1.054308851 | 2.05E-58 |
| ACACB | 23.70639638 | 2.256077536 | -3.393387819 | 3.75E-57 |
| GGT1 | 4.132783187 | 6.123528428 | 0.567249506 | 0.000226117 |
| LPL | 223.1215215 | 10.06091012 | -4.470996954 | 2.09E-56 |
| ACSL5 | 10.90343084 | 6.125013499 | -0.831997231 | 2.52E-31 |
| SLC44A4 | 7.522313724 | 40.94968671 | 2.444604033 | 1.69E-24 |
| CYP51A1 | 0.87337697 | 1.327407523 | 0.603934961 | 3.24E-09 |
| ACADM | 16.79055796 | 11.07128674 | -0.600827267 | 2.03E-21 |
| PIK3R2 | 0.158056274 | 0.366808723 | 1.214589644 | 1.50E-36 |
| EGR2 | 28.87641174 | 6.176271069 | -2.225083503 | 5.09E-39 |
| PPARGC1A | 0.680680971 | 0.322655497 | -1.076984175 | 4.90E-42 |
| DHCR24 | 99.66869297 | 187.196717 | 0.909342819 | 2.31E-16 |
| ZNF467 | 3.135862929 | 8.930472756 | 1.50987405 | 1.09E-32 |
| PNPLA2 | 107.1388694 | 33.77517635 | -1.665446769 | 3.95E-31 |
| PRKD3 | 6.58291782 | 4.322416433 | -0.606889116 | 6.80E-25 |
| INPP5J | 5.008579735 | 7.658832571 | 0.612722939 | 0.029071454 |
| MGLL | 35.43229897 | 8.4012155 | -2.076395096 | 1.02E-44 |
| PTGIS | 17.74510483 | 4.763402712 | -1.897356668 | 2.95E-37 |
| ACSS3 | 3.694357273 | 1.715538726 | -1.1066617 | 2.18E-23 |
| PCYT1B | 0.395023664 | 0.207741139 | -0.927152137 | 1.66E-19 |
| ACOT7 | 3.062994047 | 7.768716421 | 1.342733687 | 1.35E-38 |
| ARNT2 | 2.485164335 | 9.347444774 | 1.911230787 | 2.05E-22 |
| PHOSPHO1 | 0.35979384 | 0.242430762 | -0.569597714 | 5.93E-19 |
| PIAS4 | 5.337091739 | 8.621744416 | 0.691925986 | 1.40E-27 |
| OSBPL1A | 16.33262601 | 8.923637406 | -0.872052971 | 8.29E-44 |
| ADPRM | 6.212323595 | 3.816849933 | -0.702750515 | 3.51E-36 |
| FAAH2 | 4.716262198 | 8.413729939 | 0.835101585 | 4.83E-17 |
| MOGAT1 | 0.263008626 | 0.061704665 | -2.091658641 | 4.55E-37 |
| ARSH | 0.275990955 | 0.431831472 | 0.645847405 | 0.017666298 |
| ARF3 | 36.72490706 | 57.11587991 | 0.637133074 | 1.20E-31 |
| DGKG | 0.16800061 | 0.11140547 | -0.5926464 | 3.25E-20 |
| OXCT1 | 16.3176766 | 9.461560638 | -0.78628558 | 6.92E-29 |
| SLC27A1 | 7.68866482 | 4.751335073 | -0.694400136 | 5.27E-33 |
| PLD1 | 2.867792759 | 1.392092461 | -1.042685736 | 1.74E-45 |
| FABP6 | 0.046936517 | 0.903631972 | 4.266952634 | 2.31E-13 |
| CYP1A1 | 1.304714563 | 0.111163297 | -3.552981784 | 2.33E-16 |
| EBP | 8.882843901 | 18.80671131 | 1.082154046 | 2.38E-39 |
| LYPLA2 | 17.7718816 | 32.07582312 | 0.851889853 | 1.85E-35 |
| PPARG | 22.01810377 | 2.391430596 | -3.2027444 | 3.42E-55 |
| MED19 | 6.083093018 | 8.76287067 | 0.526598502 | 5.71E-22 |
| ALOX15B | 48.74792952 | 19.11932578 | -1.350309295 | 7.88E-06 |
| AMACR | 0.699810112 | 1.141130209 | 0.705428005 | 4.75E-19 |
| ACBD6 | 3.81169418 | 6.510088691 | 0.772244825 | 1.03E-41 |
| CERS6 | 7.157397748 | 15.01662431 | 1.069053476 | 2.31E-17 |
| DEGS2 | 3.383220079 | 18.34254457 | 2.438724858 | 2.30E-25 |
| HILPDA | 11.18784774 | 17.96427421 | 0.683198124 | 3.36E-11 |
| DECR2 | 3.442723045 | 6.351252568 | 0.883491016 | 1.04E-18 |
| ACSL1 | 110.6782036 | 18.50563579 | -2.580334526 | 1.31E-40 |
| FITM1 | 1.050079787 | 0.267571822 | -1.97250085 | 3.49E-05 |
| DPEP1 | 0.113163454 | 0.626552645 | 2.469027613 | 2.42E-37 |
| HSD17B2 | 0.841675131 | 0.363386672 | -1.211757982 | 4.32E-20 |
| APOA5 | 0.008056522 | 0.037577981 | 2.221658487 | 0.001595865 |
| SREBF1 | 19.29864081 | 42.81413581 | 1.149587962 | 3.27E-18 |
| TRIB3 | 4.931996931 | 14.73741285 | 1.579239475 | 2.14E-43 |
| TGFB1 | 9.803485766 | 17.33277357 | 0.822135815 | 1.86E-22 |
| ARSI | 0.575926622 | 1.533074139 | 1.41247055 | 2.79E-22 |
| NDUFAB1 | 22.0165709 | 36.95858659 | 0.747319799 | 5.07E-39 |
| ESYT3 | 0.418753957 | 0.248069087 | -0.755360858 | 2.46E-16 |
| ACAD11 | 0.236204999 | 0.111948703 | -1.077201688 | 1.37E-33 |
| MED25 | 5.314522072 | 10.02891006 | 0.916152959 | 1.93E-39 |
| RORA | 1.887171498 | 1.210465396 | -0.640663698 | 3.02E-24 |
| ABCD1 | 4.205794334 | 6.566395614 | 0.642723369 | 1.56E-10 |
| ACSL4 | 13.47229639 | 6.189951705 | -1.121995724 | 3.89E-41 |
| CPNE7 | 0.631612598 | 3.486828907 | 2.464803722 | 9.17E-25 |
| MBOAT7 | 9.907885829 | 25.38248248 | 1.357184026 | 2.88E-50 |
| CPTP | 8.670188667 | 13.23651639 | 0.610388189 | 5.74E-15 |
| ENPP6 | 0.496122199 | 0.188485444 | -1.396242399 | 3.22E-28 |
| ACOXL | 0.048985948 | 0.127765306 | 1.383056267 | 5.37E-06 |
| PLA2G2D | 0.920235299 | 2.402435013 | 1.384422703 | 0.000469969 |
| ACSM3 | 1.980978832 | 0.57807263 | -1.776890791 | 3.49E-26 |
| MTMR3 | 1.48047224 | 0.982160847 | -0.592026221 | 1.09E-34 |
| BMX | 2.867742473 | 0.476940967 | -2.58803286 | 1.34E-58 |
| CYP4F22 | 6.695532695 | 4.715123107 | -0.505903402 | 5.15E-11 |
| CD36 | 141.0590255 | 9.05513801 | -3.961418538 | 1.73E-57 |
| GPD1 | 161.5693223 | 5.179223348 | -4.963273711 | 1.89E-53 |
| GDPD5 | 5.055812342 | 2.121695628 | -1.252725209 | 1.15E-31 |
| NR1D1 | 14.0943673 | 8.957626437 | -0.653930309 | 5.93E-12 |
| KLF5 | 15.74335265 | 8.189507182 | -0.942894262 | 8.88E-18 |
| NUDT19 | 6.337045685 | 9.988831313 | 0.656505477 | 7.97E-18 |
| PLA2G4D | 0.015827007 | 0.077220671 | 2.286598625 | 7.23E-13 |
| HSD11B1 | 10.74695259 | 2.304753774 | -2.221243091 | 1.06E-30 |
| SGPL1 | 10.74346344 | 17.75068447 | 0.724415497 | 1.11E-26 |
| ACADS | 16.85032497 | 7.239955324 | -1.218723715 | 3.24E-33 |
| LPIN1 | 6.51334091 | 3.382568691 | -0.945278508 | 6.38E-35 |
| STARD3 | 7.321712856 | 16.83325601 | 1.20106116 | 1.81E-11 |
| PLA2G5 | 1.055911246 | 0.421305658 | -1.325549379 | 3.94E-33 |
| MTMR8 | 0.388344436 | 0.227621891 | -0.770697482 | 7.67E-26 |
| NEU1 | 13.04305901 | 22.12126726 | 0.762151769 | 1.52E-34 |
| MLYCD | 1.352350743 | 0.744342551 | -0.861430759 | 1.79E-45 |
| CH25H | 3.465340546 | 1.636675844 | -1.082228522 | 5.95E-17 |
| HPGDS | 2.602606622 | 1.433821322 | -0.860092016 | 1.87E-14 |
| LEP | 96.68836343 | 1.682883019 | -5.84433547 | 7.66E-54 |
| FABP9 | 0.183182422 | 0.025717435 | -2.832462405 | 3.26E-44 |
| GGPS1 | 11.6346324 | 20.38820681 | 0.809309264 | 5.47E-42 |
| ENPP7 | 0.00570619 | 0.020638787 | 1.854758511 | 1.30E-07 |
| PLA2R1 | 6.106837005 | 2.08561694 | -1.549951133 | 2.18E-46 |
| EBF1 | 14.67090861 | 1.919861753 | -2.933883891 | 2.88E-59 |
| GDPD1 | 1.316598823 | 2.487788865 | 0.918048238 | 3.79E-09 |
| OSBPL9 | 13.4577585 | 9.288668374 | -0.534894445 | 5.44E-27 |
| STS | 9.245327541 | 4.505266991 | -1.037111826 | 2.34E-29 |
| CYP27B1 | 0.493718983 | 1.278662833 | 1.372873874 | 1.60E-10 |
| CERS1 | 0.098828797 | 0.210124524 | 1.088241174 | 0.049353658 |
| APOA1 | 0.060991907 | 0.810062019 | 3.731342639 | 6.44E-09 |
| SQLE | 5.664515642 | 26.03950154 | 2.200677326 | 2.80E-49 |
| EHHADH | 4.912002315 | 2.576324809 | -0.930996749 | 6.53E-32 |
| PLA2G2A | 28.11119648 | 12.14607147 | -1.210655096 | 3.12E-15 |
| SLCO1A2 | 0.412704926 | 0.068491619 | -2.591111283 | 2.03E-34 |
| MBOAT2 | 4.291976018 | 11.28028847 | 1.394090043 | 1.20E-29 |
| PLIN3 | 19.31496999 | 29.17047468 | 0.594789427 | 6.99E-25 |
| HSD17B11 | 21.39280371 | 8.962520093 | -1.255149219 | 5.89E-44 |
| PIK3R6 | 0.447279271 | 0.710258485 | 0.667168263 | 1.28E-14 |
| MFSD2A | 1.444073291 | 2.564134202 | 0.828327807 | 0.000123538 |
| SRD5A3 | 6.189314288 | 11.89320041 | 0.942285502 | 6.97E-27 |
| ETNK2 | 4.788197175 | 12.69197644 | 1.40636228 | 3.09E-18 |
| SEC24D | 6.002739892 | 10.71306895 | 0.835678765 | 5.23E-25 |
| THRSP | 79.55585219 | 12.52765834 | -2.666851288 | 5.71E-43 |
| LPCAT1 | 7.04822361 | 16.58845961 | 1.234848325 | 3.69E-45 |
| SCD | 376.3800916 | 141.0466041 | -1.416018389 | 1.53E-05 |
| INPP5K | 12.15092123 | 8.252309039 | -0.558195942 | 3.51E-27 |
| DGKB | 0.045691415 | 0.023477152 | -0.96066571 | 4.83E-28 |
| ADIPOQ | 196.103717 | 6.692548672 | -4.872917345 | 1.79E-49 |
| CARM1 | 7.546769604 | 11.40295068 | 0.595476055 | 1.73E-29 |
| PPT1 | 60.23956828 | 97.12681665 | 0.689158247 | 1.06E-17 |
| FDXR | 2.266676102 | 5.223563466 | 1.204456085 | 1.01E-23 |
| PPARGC1B | 0.907796854 | 0.483352384 | -0.909294132 | 1.85E-32 |
| B3GALNT1 | 4.899648075 | 8.019053568 | 0.710753847 | 8.58E-17 |
| AGT | 3.104572233 | 10.53214182 | 1.76233245 | 0.003619763 |
| SLC27A2 | 3.371289522 | 7.822854101 | 1.214394529 | 0.000215544 |
| ACSBG2 | 0.147536136 | 0.084685334 | -0.80088431 | 8.89E-19 |
| HSD17B7 | 1.669209328 | 3.877807793 | 1.216076409 | 2.42E-41 |
| HELZ2 | 2.708455887 | 6.081336924 | 1.166917927 | 5.73E-23 |
| CPNE1 | 22.50976407 | 35.38476794 | 0.652577522 | 3.10E-14 |
| PTEN | 14.75964622 | 9.596148165 | -0.621130803 | 8.47E-32 |
| LYPLA1 | 12.1959421 | 23.90964014 | 0.97119121 | 7.50E-26 |
| SMPD3 | 0.853223219 | 1.556893082 | 0.86767474 | 0.01332647 |
| AHR | 24.05622932 | 15.34622916 | -0.648526323 | 3.98E-19 |
| CERS2 | 31.18900833 | 77.14627111 | 1.306558743 | 3.28E-46 |
| PIK3R1 | 37.49894546 | 14.14799628 | -1.40625228 | 4.33E-45 |
| CERS4 | 12.28615793 | 22.43607909 | 0.868786741 | 2.35E-15 |
| ECI1 | 9.535027919 | 18.31594906 | 0.94179139 | 7.86E-24 |
| MTMR10 | 6.799812667 | 3.395752987 | -1.001763482 | 3.56E-48 |
| ACOT4 | 3.124992769 | 6.065824073 | 0.956850804 | 1.11E-14 |
| ME1 | 17.92542254 | 6.492318873 | -1.465201361 | 4.59E-16 |
| HADHA | 88.41421982 | 61.11264236 | -0.532807558 | 1.76E-45 |
| ACADVL | 65.54121649 | 41.73831807 | -0.651029985 | 1.33E-29 |
| SLC25A20 | 14.8554595 | 9.510938954 | -0.64333355 | 1.94E-29 |
| PLB1 | 0.696661517 | 0.487134123 | -0.516138826 | 1.09E-25 |
| PMVK | 29.90833688 | 55.14565093 | 0.882699423 | 9.59E-26 |
| PTGDS | 22.12152199 | 10.53708094 | -1.069975392 | 1.77E-29 |
| GLA | 16.03291766 | 25.40127344 | 0.663863836 | 0.000116351 |
| ARV1 | 9.465422423 | 16.48394692 | 0.800322926 | 9.15E-32 |
| CAV1 | 185.9221896 | 19.74363849 | -3.235239176 | 2.69E-64 |
| GPD1L | 27.06252297 | 18.09465991 | -0.580732352 | 2.12E-17 |
| HMGCLL1 | 0.837729822 | 0.459502865 | -0.866411176 | 2.41E-27 |
| WNT10B | 0.631082547 | 0.290998535 | -1.116816835 | 1.74E-16 |
| CYP7A1 | 0.059081541 | 0.017961211 | -1.71782285 | 1.91E-44 |
| DGAT2 | 40.82901222 | 4.627586104 | -3.141262925 | 3.15E-37 |
| HSD17B14 | 4.272644095 | 7.01842182 | 0.716017511 | 0.000455034 |
| TNFAIP8L1 | 2.143823192 | 3.669180926 | 0.775272118 | 2.27E-26 |
| CEBPA | 26.07416623 | 6.503707394 | -2.003286864 | 4.91E-22 |
| ABCB11 | 0.10943499 | 0.034537194 | -1.663851326 | 3.56E-23 |
| SPHK1 | 4.43031826 | 6.863257597 | 0.631483163 | 0.000314575 |
| MAPKAPK2 | 30.9949036 | 65.54178357 | 1.080383916 | 9.36E-46 |
| PIP5K1B | 2.264099907 | 1.112023192 | -1.025750744 | 3.28E-32 |
| ACADL | 2.43522018 | 0.269392539 | -3.176270422 | 9.29E-54 |
| ABHD3 | 3.771565414 | 6.967362568 | 0.885449192 | 1.31E-16 |
| CYP46A1 | 0.275790167 | 0.123007238 | -1.164827815 | 2.42E-40 |
| LCLAT1 | 1.841469841 | 3.038036538 | 0.722281452 | 4.08E-23 |
| PNPLA7 | 2.867055812 | 1.543463803 | -0.89339834 | 5.78E-31 |
| SMPD4 | 7.277048631 | 11.1392138 | 0.614222055 | 3.23E-31 |
| PIKFYVE | 5.521508009 | 3.694559158 | -0.579660114 | 4.41E-24 |
| OSBPL6 | 0.732837093 | 1.19920335 | 0.710511886 | 0.001696974 |
| SBF2 | 4.691299967 | 2.62642124 | -0.836889429 | 2.46E-32 |
| CSNK1G2 | 10.5744317 | 15.20032961 | 0.523522477 | 1.98E-14 |
| CIDEA | 39.47711932 | 1.582100257 | -4.641103788 | 1.44E-48 |
| CHD9 | 6.248997523 | 3.366839593 | -0.89222978 | 9.04E-34 |
| FADS2 | 6.168116102 | 17.9654156 | 1.542320484 | 2.24E-08 |
| NR1H3 | 9.692345741 | 5.929574709 | -0.708917236 | 6.31E-26 |
| HSD17B13 | 3.606702015 | 0.121960099 | -4.886199104 | 1.01E-58 |
| FA2H | 1.14229296 | 2.009397807 | 0.814830507 | 0.001258029 |
| PLIN2 | 23.17835513 | 12.80313861 | -0.856280666 | 2.77E-23 |
| ELOVL7 | 4.444345682 | 2.481683263 | -0.840652038 | 7.55E-13 |
| SMPD2 | 4.842834198 | 8.298858735 | 0.777061339 | 3.02E-23 |
| TBL1XR1 | 14.247697 | 20.51677442 | 0.526075192 | 3.67E-14 |
| PIK3C2G | 5.134393399 | 0.759957762 | -2.756202699 | 2.30E-36 |
| NEU4 | 0.024547453 | 0.211917489 | 3.109857382 | 1.26E-06 |
| ACAA2 | 20.31357227 | 9.694324449 | -1.067231697 | 1.94E-39 |
| ACAT2 | 4.175787972 | 6.588274478 | 0.657852201 | 2.40E-13 |
| PLEKHA4 | 16.59002368 | 8.736885757 | -0.925124915 | 1.96E-23 |
| NCOA1 | 13.78585644 | 9.607288369 | -0.5209877 | 4.44E-26 |
| NFYA | 11.02753252 | 16.39679961 | 0.572304236 | 1.26E-15 |
| GK | 0.870550506 | 1.863029106 | 1.097650308 | 7.08E-30 |
| TNFRSF21 | 23.83440652 | 14.69788969 | -0.697436674 | 1.12E-24 |
| HSD3B7 | 7.147278306 | 10.77460215 | 0.592168727 | 2.46E-12 |
| STARD10 | 14.87764182 | 61.18221688 | 2.039966512 | 1.83E-32 |
| CYP4A11 | 0.153818834 | 0.086760604 | -0.826120157 | 1.47E-16 |
| BDH2 | 12.05833798 | 6.963094828 | -0.792230496 | 5.76E-37 |
| AKR1C3 | 18.87509049 | 3.947522819 | -2.25746405 | 5.86E-48 |
| SC5D | 12.32566833 | 7.510093443 | -0.714763112 | 1.39E-22 |
| GBA | 10.96415193 | 20.44607128 | 0.899029431 | 6.43E-39 |
| ACOT8 | 2.931008018 | 4.582226225 | 0.644651772 | 1.47E-27 |
| CIDEC | 144.1131337 | 4.57002629 | -4.978855546 | 4.70E-52 |
| SRD5A2 | 0.037257407 | 0.185534896 | 2.316091404 | 2.09E-20 |
| SAMD8 | 9.114170883 | 5.893742583 | -0.62892737 | 2.48E-29 |
| AGMO | 0.601213467 | 0.134827591 | -2.156761562 | 8.39E-50 |
| G0S2 | 227.3218866 | 16.14258503 | -3.815793062 | 3.31E-46 |
| AGPAT2 | 92.36490749 | 31.46508286 | -1.553593086 | 5.61E-14 |
| PITPNM1 | 2.839033909 | 4.429399189 | 0.641710942 | 2.35E-09 |
| MED24 | 10.73837293 | 17.87218245 | 0.734940405 | 1.38E-06 |
| HPGD | 7.371024168 | 3.587417304 | -1.038919513 | 3.44E-16 |
| PTGES2 | 7.97295209 | 13.26847648 | 0.734816821 | 4.65E-25 |
| CRAT | 17.01290045 | 34.9593236 | 1.039048151 | 1.96E-05 |
| GLB1 | 12.28702877 | 19.29342811 | 0.65097342 | 5.12E-33 |
| PECR | 7.427112279 | 3.316569398 | -1.163109674 | 5.08E-12 |
| NSDHL | 10.93178305 | 15.82999276 | 0.534131862 | 9.61E-23 |
| CYP21A2 | 0.238684434 | 1.171381769 | 2.295034955 | 5.81E-17 |
| CEBPD | 27.2696006 | 18.99581836 | -0.521611704 | 6.14E-13 |
| INPPL1 | 13.23335205 | 19.35263855 | 0.54835173 | 9.24E-17 |
| FDPS | 16.1309781 | 26.40202334 | 0.710814577 | 7.49E-28 |
| ACER2 | 4.140281305 | 2.644420308 | -0.646777294 | 4.35E-17 |

294 differential genes were choose through |logFC| > 0.5; *p* < 0.05

**Supplementary table 3 Differential genes between the high-risk group and low-risk group**

| gene | lowMean | highMean | logFC | pValue | fdr |
| --- | --- | --- | --- | --- | --- |
| KRT4 | 3.541546401 | 1.241276042 | -1.512555458 | 0.008955941 | 0.012060991 |
| IGKV5-2 | 9.742883518 | 3.166777814 | -1.621333168 | 6.80E-11 | 2.16E-10 |
| OPRPN | 12.45179695 | 3.230246792 | -1.946637659 | 3.23E-08 | 7.83E-08 |
| IRX1 | 8.585218342 | 2.832927607 | -1.59956109 | 5.14E-33 | 2.96E-31 |
| ABCA12 | 1.463504203 | 4.458422454 | 1.607106435 | 2.05E-15 | 1.05E-14 |
| CD79B | 5.368939954 | 1.650740571 | -1.701523865 | 2.15E-46 | 1.92E-43 |
| GSTA1 | 6.571493622 | 1.87677944 | -1.807962201 | 1.96E-13 | 8.09E-13 |
| VPREB3 | 2.742335634 | 0.939106268 | -1.546044828 | 4.17E-29 | 1.26E-27 |
| ZAP70 | 1.962779416 | 0.679442021 | -1.530475695 | 1.63E-33 | 9.90E-32 |
| CD52 | 50.14731297 | 17.20219617 | -1.543579636 | 2.50E-20 | 2.25E-19 |
| MUC2 | 1.085586292 | 3.083342582 | 1.506020784 | 0.001622099 | 0.002388839 |
| KRTDAP | 7.519996258 | 1.031471601 | -2.866027844 | 6.38E-12 | 2.25E-11 |
| MED15P4 | 0.684393218 | 2.248285198 | 1.715927686 | 0.031622873 | 0.039626941 |
| IGHV3-73 | 68.12845488 | 13.05048617 | -2.384153935 | 5.81E-13 | 2.28E-12 |
| IGHA2 | 240.2707603 | 84.43134643 | -1.508810464 | 1.72E-24 | 2.68E-23 |
| IGLV1-36 | 18.98597588 | 5.264832555 | -1.850474603 | 5.14E-15 | 2.52E-14 |
| RRAD | 2.108995985 | 0.715860785 | -1.558805392 | 2.80E-32 | 1.40E-30 |
| OSR1 | 2.421486996 | 0.814962582 | -1.571087528 | 1.01E-32 | 5.41E-31 |
| KLK10 | 7.509800704 | 2.313556491 | -1.698662295 | 1.15E-22 | 1.39E-21 |
| CYP2A6 | 9.278456308 | 37.45759949 | 2.013301742 | 0.002185978 | 0.003167032 |
| IGHV2-70D | 9.731734337 | 3.231542551 | -1.590473949 | 2.63E-14 | 1.19E-13 |
| TNNI2 | 2.961259985 | 0.731259286 | -2.017756214 | 2.01E-38 | 3.13E-36 |
| CEACAM6 | 30.87495159 | 111.1926486 | 1.848552629 | 1.56E-18 | 1.12E-17 |
| IGKV1D-43 | 3.374990317 | 0.435638857 | -2.953678815 | 8.61E-14 | 3.70E-13 |
| MIA | 5.308612321 | 1.742379201 | -1.60727615 | 3.98E-37 | 4.76E-35 |
| IGLV3-12 | 2.243268982 | 0.486119677 | -2.206219182 | 1.32E-12 | 4.99E-12 |
| SNORA23 | 0.231738751 | 12.80677548 | 5.788264157 | 0.000201907 | 0.00032843 |
| CXCL1 | 2.924356314 | 0.882608551 | -1.728273475 | 7.42E-24 | 1.06E-22 |
| IGHD | 10.26838719 | 3.25878524 | -1.655803418 | 8.37E-18 | 5.48E-17 |
| SNORA79B | 0.223501889 | 2.572142849 | 3.52461184 | 0.032417197 | 0.040565402 |
| IGLV8-61 | 56.12285411 | 19.7043521 | -1.510074066 | 1.14E-12 | 4.33E-12 |
| IGLV3-21 | 191.9745331 | 53.51091179 | -1.843009922 | 1.50E-12 | 5.63E-12 |
| IGLC7 | 20.90479416 | 6.714489965 | -1.638484116 | 1.40E-14 | 6.58E-14 |
| RN7SL3 | 0.568180691 | 58.51679053 | 6.686357029 | 0.000675476 | 0.001036344 |
| FOXCUT | 3.374319907 | 1.160593983 | -1.539733403 | 8.75E-19 | 6.48E-18 |
| AC010970.1 | 0.564303036 | 4.857818892 | 3.105766688 | 0.000586615 | 0.000905089 |
| IGHV6-1 | 7.961635394 | 2.38819351 | -1.737145064 | 7.57E-10 | 2.16E-09 |
| IGLV3-10 | 77.66008205 | 24.08369872 | -1.689116257 | 4.42E-10 | 1.30E-09 |
| ECEL1 | 6.211643073 | 0.602756184 | -3.365328481 | 2.33E-15 | 1.18E-14 |
| CHRDL2 | 2.588420403 | 0.63214678 | -2.033740469 | 2.77E-14 | 1.25E-13 |
| IGLV4-69 | 71.30504775 | 23.46555578 | -1.603459579 | 5.27E-15 | 2.58E-14 |
| IGKC | 1137.219851 | 368.4460202 | -1.625986012 | 6.16E-21 | 5.95E-20 |
| MYBPC1 | 10.68746134 | 3.383776635 | -1.659212958 | 5.11E-18 | 3.44E-17 |
| IGLC6 | 2.132780214 | 0.546671555 | -1.963989087 | 1.11E-20 | 1.04E-19 |
| IGLVI-70 | 4.840238338 | 0.473328366 | -3.354164801 | 8.07E-17 | 4.77E-16 |
| KRT81 | 73.50368664 | 20.22225843 | -1.861872484 | 3.82E-17 | 2.33E-16 |
| IGHG1 | 978.0341585 | 291.9876735 | -1.743977387 | 2.02E-12 | 7.47E-12 |
| KRT17 | 103.9106869 | 31.85515772 | -1.705745153 | 3.56E-31 | 1.49E-29 |
| CXCL2 | 2.694510988 | 0.944106834 | -1.513001442 | 1.11E-28 | 3.16E-27 |
| IGHV3-20 | 6.312776363 | 2.202205128 | -1.519325786 | 2.08E-14 | 9.52E-14 |
| IGKV1-33 | 1.915758976 | 0.546079568 | -1.810732982 | 1.28E-12 | 4.82E-12 |
| IGHV1OR15-2 | 4.258677353 | 1.160832677 | -1.875245395 | 1.53E-12 | 5.74E-12 |
| IGKV6D-21 | 5.742508981 | 1.201492476 | -2.256853593 | 1.19E-13 | 5.02E-13 |
| VMO1 | 8.744169526 | 1.817271563 | -2.266547347 | 1.23E-19 | 1.02E-18 |
| ACTG2 | 26.17245417 | 8.971032665 | -1.544703241 | 3.28E-12 | 1.19E-11 |
| DES | 5.788353019 | 1.29394648 | -2.161374965 | 5.30E-11 | 1.70E-10 |
| AP000851.1 | 3.789054779 | 0.696678503 | -2.443273045 | 1.79E-27 | 4.41E-26 |
| CPLX2 | 0.387173663 | 2.137742032 | 2.465035047 | 4.61E-05 | 8.08E-05 |
| IGKV2D-24 | 2.374555443 | 0.66659278 | -1.832779846 | 2.38E-12 | 8.72E-12 |
| S1PR4 | 2.314609794 | 0.767636087 | -1.592274559 | 6.89E-42 | 2.20E-39 |
| KRT1 | 15.4682955 | 0.745023618 | -4.375884259 | 0.000137072 | 0.000227525 |
| HRCT1 | 7.506127858 | 2.196757147 | -1.772693477 | 2.32E-24 | 3.54E-23 |
| ASCL1 | 1.333609427 | 3.907453614 | 1.550892539 | 0.000602012 | 0.000927883 |
| IGLV2-14 | 319.7638871 | 108.0783925 | -1.564928893 | 9.63E-14 | 4.11E-13 |
| WNT6 | 1.772391484 | 0.508647092 | -1.800960358 | 3.96E-40 | 8.69E-38 |
| LOR | 2.469806489 | 0.028814013 | -6.421483604 | 5.95E-37 | 6.81E-35 |
| SNORD17 | 1.314396821 | 46.31272139 | 5.138935732 | 0.001324043 | 0.001966542 |
| IGKV3D-15 | 6.919623217 | 2.038260213 | -1.763355239 | 1.15E-15 | 6.03E-15 |
| MS4A1 | 3.751280814 | 1.208419182 | -1.634262274 | 8.18E-20 | 6.89E-19 |
| SNORA22 | 0.406371214 | 2.433588415 | 2.582215075 | 0.025947773 | 0.032959354 |
| AF178030.1 | 0.195549354 | 1.842732321 | 3.236241841 | 0.008615621 | 0.011628398 |
| CRISP3 | 15.41769147 | 45.04658432 | 1.546830954 | 2.97E-06 | 5.90E-06 |
| IGKV2OR22-4 | 1.981341298 | 0.531760658 | -1.897628466 | 1.96E-13 | 8.09E-13 |
| PI3 | 31.15785157 | 6.291374351 | -2.308148645 | 8.86E-14 | 3.80E-13 |
| LTB | 13.05824226 | 3.057952801 | -2.094322668 | 2.26E-41 | 6.29E-39 |
| GZMM | 3.179107077 | 0.936837428 | -1.762750989 | 9.13E-43 | 3.72E-40 |
| SPIB | 2.805120731 | 0.517703212 | -2.437865692 | 4.29E-35 | 3.32E-33 |
| C2orf40 | 4.13658762 | 0.960198448 | -2.107036632 | 3.09E-20 | 2.74E-19 |
| MSLN | 18.26052633 | 4.926230731 | -1.890172243 | 3.86E-13 | 1.55E-12 |
| C4BPA | 2.032330842 | 0.342972047 | -2.566972375 | 2.90E-13 | 1.18E-12 |
| IGLV3-9 | 20.22580835 | 4.910419887 | -2.042279064 | 3.72E-15 | 1.85E-14 |
| PTGDS | 16.31357226 | 5.185232282 | -1.653592207 | 2.84E-37 | 3.62E-35 |
| IGLV1-41 | 7.993905679 | 0.917091372 | -3.123763165 | 1.10E-11 | 3.79E-11 |
| SNORC | 2.699274821 | 0.660724682 | -2.030450726 | 1.23E-31 | 5.57E-30 |
| OLFM4 | 42.62456433 | 8.792443758 | -2.277348982 | 6.23E-13 | 2.44E-12 |
| IGHV4-39 | 168.6883075 | 52.40212304 | -1.68666281 | 1.66E-14 | 7.70E-14 |
| IGKV2D-28 | 1.726384828 | 0.311717792 | -2.469441684 | 5.89E-16 | 3.18E-15 |
| SNORA14B | 0.633399201 | 2.458535432 | 1.956612194 | 6.83E-05 | 0.000117567 |
| CEACAM5 | 7.495378614 | 22.94924491 | 1.614373426 | 5.37E-26 | 1.07E-24 |
| IGLV9-49 | 15.66225667 | 3.986678424 | -1.974032953 | 2.22E-11 | 7.41E-11 |
| OR2I1P | 7.88401914 | 2.559193422 | -1.623242089 | 1.54E-22 | 1.82E-21 |
| C6orf15 | 7.394779555 | 0.815490089 | -3.180767892 | 2.90E-16 | 1.62E-15 |
| PHKG1 | 2.057908951 | 0.589846647 | -1.802767328 | 3.10E-16 | 1.72E-15 |
| SYT8 | 2.826684777 | 0.572225669 | -2.304454888 | 4.47E-36 | 4.22E-34 |
| IGKV3D-20 | 17.46893798 | 5.316164838 | -1.716334158 | 2.54E-17 | 1.58E-16 |
| IGKV2D-29 | 19.59588191 | 6.66136551 | -1.556660653 | 5.31E-13 | 2.10E-12 |
| IGKV1D-27 | 5.222546005 | 0.557296795 | -3.228235535 | 1.74E-11 | 5.86E-11 |
| TRPA1 | 0.910218161 | 4.620621523 | 2.343802646 | 0.02907152 | 0.036669914 |
| IGHV3OR16-9 | 1.841628772 | 0.639115637 | -1.526833387 | 9.31E-15 | 4.43E-14 |
| IGHD3-9 | 2.506790388 | 0.720960311 | -1.79784962 | 5.70E-08 | 1.35E-07 |
| MALAT1 | 22.29029514 | 107.3827115 | 2.268274115 | 0.001458413 | 0.002157981 |
| CCL19 | 47.92334277 | 13.33721669 | -1.845270917 | 1.76E-34 | 1.24E-32 |
| LINC01522 | 1.473617369 | 4.490031736 | 1.60736367 | 3.37E-08 | 8.16E-08 |
| SBSN | 4.399712736 | 1.486424393 | -1.565563248 | 0.029000728 | 0.036584063 |
| CD79A | 15.5514154 | 4.798952877 | -1.696254341 | 2.01E-28 | 5.59E-27 |
| CHI3L1 | 52.23199469 | 17.82185605 | -1.551286206 | 3.62E-19 | 2.80E-18 |
| IGLV4-3 | 1.645368122 | 0.535022675 | -1.620738458 | 4.09E-08 | 9.80E-08 |
| AC026355.1 | 0.501163619 | 2.11982098 | 2.080588839 | 1.17E-06 | 2.44E-06 |
| RHCG | 3.19953029 | 0.902138327 | -1.826439558 | 2.34E-08 | 5.77E-08 |
| GLYATL1P4 | 0.427979981 | 3.444191536 | 3.008550156 | 0.000175501 | 0.000287431 |
| IGKV6-21 | 17.36940303 | 4.786207818 | -1.859593225 | 6.02E-15 | 2.92E-14 |
| KRT14 | 112.3714629 | 36.60830321 | -1.618032893 | 2.30E-28 | 6.38E-27 |
| PLA2G2D | 3.862894523 | 1.055899328 | -1.871209994 | 6.18E-20 | 5.29E-19 |
| MYOC | 2.43706661 | 0.145821427 | -4.062871053 | 8.19E-05 | 0.000139576 |
| IGKV1OR2-6 | 2.299881721 | 0.696088586 | -1.724216844 | 1.44E-15 | 7.48E-15 |
| IGHJ3P | 7.072638134 | 2.321379698 | -1.607265932 | 4.90E-16 | 2.67E-15 |
| LINC00578 | 0.721255962 | 2.373806261 | 1.718618949 | 7.42E-12 | 2.60E-11 |
| LGALS7B | 4.697286367 | 1.414933975 | -1.731092815 | 4.84E-15 | 2.38E-14 |
| ARHGAP40 | 7.137373988 | 2.286915359 | -1.641990398 | 3.60E-14 | 1.60E-13 |

23 up-regulated and 97 down-regulated differential genes (DEGs) between the high-risk group and low-risk group were choose through |logFC|>1.5; *p* < 0.05.

**Supplementary table 4 Eight small molecule drugs**

| cmap name | enrichment | p Value |
| --- | --- | --- |
| rifampicin | -0.814 | 0.00221 |
| Prestwick-682 | -0.781 | 0.00465 |
| clenbuterol | -0.678 | 0.00811 |
| anisomycin | -0.738 | 0.00937 |
| fusaric acid | -0.699 | 0.01725 |
| withaferin A | -0.667 | 0.02777 |
| spironolactone | -0.603 | 0.02778 |
| MG-262 | -0.755 | 0.03009 |
| desipramine | -0.66 | 0.03046 |

The eight small molecule drugs were screened based on DEGs in high- and low- risk groups from the CMap database (https://portals.broadinstitute.org/cmap/).
